# Seven new species of Selaginella subg. Stachygynandrum (Selaginellaceae) from Brazil and new synonyms for the genus

**DOI:** 10.3897/phytokeys.50.4873

**Published:** 2015-06-16

**Authors:** Iván A. Valdespino, Gustavo Heringer, Alexandre Salino, Luiz A. de Araújo Góes-Neto, Jorge Ceballos

**Affiliations:** 1 Departamento de Botánica, Facultad de Ciencias Naturales, Exactas y Tecnología, Universidad de Panamá, Apartado Postal 0824-00073, Panama; 2 Pós-Graduação em Botânica, Departamento de Biologia Vegetal, Universidade Federal de Viçosa, CEP 36.570-000 Viçosa, Minas Gerais, Brazil; 3 Departamento de Botânica, Instituto de Ciências Biológicas, Universidade Federal de Minas Gerais, CP 486, 31270-901, Belo Horizonte, MG, Brazil; 4 Smithsonian Tropical Research Institute, Apartado Postal 0843-03092, Panama

**Keywords:** Atlantic Rainforest, Chapada Diamantina, Chapada dos Guimarães, Espinhaço Mountain Range, Mucugê, Serra do Sincorá

## Abstract

We describe seven new species of Selaginella subg. Stachygynandrum (*Selaginella alstonii*, *Selaginella blepharodella*, *Selaginella crinita*, *Selaginella mucronata*, *Selaginella mucugensis*, *Selaginella saltuicola*, and *Selaginella sematophylla*) from Brazil and discuss their possible affinities and conservation status. Scanning electron micrographs of stem sections, leaves, and spores are provided to illustrate the new taxa. In *Selaginella alstonii* and *Selaginella saltuicola* vegetative growth from strobilus tips is reported and discussed. Four of the new species are from the Espinhaço Mountain Range associated with Campos Rupestres (montane savannah/rocky fields) vegetation. Three of these (i.e., *Selaginella blepharodella*, *Selaginella crinita*, and *Selaginella mucugensis*) were collected in the northern part of the range in Chapada Diamantina, state of Bahia, while *Selaginella alstonii* is from the southern part of the range in the state of Minas Gerais. *Selaginella mucronata* is found in Atlantic Rainforest vegetation in the state of Espírito Santo, whereas *Selaginella saltuicola* inhabits Cerrado (tropical savannah) vegetation in the state of Mato Grosso. *Selaginella sematophylla* is the most widely distributed of the new species and was collected in Espírito Santo, Minas Gerais, and Rio de Janeiro states in Campos Rupestres and Atlantic Rainforest vegetation. *Selaginella alstonii* occurs in rocky caves, *Selaginella blepharodella*, *Selaginella crinita*, *Selaginella mucugensis*, and *Selaginella sematophylla* seem adapted to seasonally dry places, living on sandy or humid soils, *Selaginella mucronata* occupies humid, forest understory, and *Selaginella saltuicola* is adapted to wet places associated with rocks or logs in waterfalls. Of the seven new species, six are considered local endemics (except for *Selaginella sematophylla*) because of their restricted currently known distributions to one or two localities within a single state in Brazil. Additionally, we propose new synonymy for *Selaginella palmiformis* (syn. = Selaginella bahiensis subsp. manausensis, ≡ *Selaginella manausensis*) and *Selaginella vestiens* (syn. = *Selaginella fragillima*); the last species is endemic to Brazil, recorded in the states of Goiás and Minas Gerais. Finally, based on literature discussed and this study, we conclude that the number of well-documented Brazilian *Selaginella* species is 61, of which 58 are native and three introduced and naturalized. These statistics are likely to change with further work on *Selaginella* from Brazil.

## Abstract (es)

Describimos siete nuevas especies de Selaginella subg. Stachygynandrum (*Selaginella alstonii*, *Selaginella blepharodella*, *Selaginella crinita*, *Selaginella mucronata*, *Selaginella mucugensis*, *Selaginella saltuicola* y *Selaginella sematophylla*) de Brasil y discutimos sus posibles afinidades y estado de conservación. Micrografias electrónicas de barrido de secciones de los tallos, hojas y esporas se proveen para ilustrar los nuevos taxa. Igualmente, se describe y discute el crecimiento vegetativo a partir del ápice de los estróbilos en *Selaginella alstonii* y *Selaginella saltuicola*. Cuatro de las especies nuevas proceden de la Cadena del Espinhaço asociadas a vegetación de Campos Rupestres (sabana montana). Tres de éstas (i.e., *Selaginella blepharodella*, *Selaginella crinita* y *Selaginella mucugensis*) fueron recolectadas en la parte norteña de la Cadena del Espinhaço en la Chapada Diamantina, estado de Bahia, mientras que *Selaginella alstonii* se registra para la parte sureña en el estado de Minas Gerais. *Selaginella mucronata* se encuentra en vegetación de Bosques Lluviosos del Atlántico en el estado de Espírito Santo, mientras que *Selaginella saltuicola* habita vegetación de Cerrado (sabana tropical) en el estado de Mato Grosso. De las nuevas especies, *Selaginella sematophylla* es la más ampliamente distribuida y se ha recolectada en los estados de Espírito Santo, Minas Gerais y Río de Janeiro en vegetación de Campos Rupestres y Bosques Lluviosos del Atlántico. *Selaginella alstonii* crece sobre rocas en cuevas, mientras que *Selaginella blepharodella*, *Selaginella crinita*, *Selaginella mucugensis* y *Selaginella sematophylla* parecen estar adaptadas a lugares estacionalmente secos, creciendo sobre suelos arenosos o húmedos; a su vez, *Selaginella mucronata* crece en el sotobosque de bosques húmedos y *Selaginella saltuicola* está adaptada a vivir en lugares húmedos asociada a rocas o troncos en cascadas. De las siete nuevas especies, seis son consideradas tentativamente endémicas locales (con la excepción de *Selaginella sematophylla*) debido a su distribución restringida a una o dos localidades dentro de un sólo estado de Brasil. Adicionalmente, proponemos nuevos sinónimos para *Selaginella palmiformis* (syn. = Selaginella bahiensis subsp. manausensis, ≡ *Selaginella manausensis*) y *Selaginella vestiens* (syn. = *Selaginella fragillima*), la cual se confirma como endémica de Brasil donde se registra para los estados de Goiás y Minas Gerais. Finalmente, de acuerdo con este estudio y la literatura discutida, estimamos que el número de especies brasileñas de *Selaginella* debidamente documentadas es de 61, de las cuales 58 son nativas y tres introducidas y naturalizadas. Esta estadística muy probablemente cambiará conforme se realicen estudios adicionales sobre *Selaginella* en Brasil.

## Introduction

The genus *Selaginella* P. Beauv. (Selaginellaceae) is cosmopolitan and comprises 600–750 species mostly distributed in tropical and subtropical regions of the world, although some are adapted to live in dry, desert-like areas and some are circumboreal (Jermy 1990, Valdespino 1993a, Mickel et al. 2004).

Alston et al. (1981) recorded 45 species and two subspecies of *Selaginella* from Brazil, while Hirai (2015) listed 56 taxa, including two subspecies and three introduced species. As part of ongoing work on *Selaginella* by the senior author and a study of this genus in the state of Minas Gerais conducted by Heringer (2011) under the supervision of Salino, we now describe seven new taxa from Brazil: *Selaginella alstonii* G. Heringer, Salino & Valdespino, *Selaginella blepharodella* Valdespino, *Selaginella crinita* Valdespino, *Selaginella mucronata* G. Heringer, Salino & Valdespino, *Selaginella mucugensis* Valdespino, *Selaginella saltuicola* Valdespino, and *Selaginella sematophylla* Valdespino, G. Heringer & Salino, and place them in subg. *Stachygynandrum* (P. Beauv.) Baker following Jermy’s (1986, 1990) infrageneric classification.

Three of the new species *Selaginella blepharodella*, *Selaginella crinita*, and *Selaginella mucugensis* are reported from three localities (i.e., Pico das Almas in Serra do Rio de Contas and Ibicoara and Mucugê in Serra do Sincorá) of Chapada Diamantina in the state of Bahia, whereas *Selaginella alstonii* was collected in Santo Antônio do Itambé in the state of Minas Gerais. These localities are within the Espinhaço Mountain Range, which is dominated by “Campos Rupestres” (montane savannah/rocky fields) vegetation (Melo 2000, São-Pedro and Feio 2011) and recognized as an important biodiversity and endemism center (Harley and Simmons 1986, Melo 2000, Rapini et al. 2008, Bünger et al. 2014). *Selaginella mucronata* was collected in Castelo, Parque Estadual do Forno Grande, a locality that has highland remnants of the rich, biodiverse Atlantic Rainforest vegetation in the state of Espírito Santo, southeastern Brazil (Meirelles and Goldenberg 2012, Silva-Soares and Scherrer 2013). *Selaginella saltuicola* is recorded from Chapada dos Guimarães, a high plateau in the state of Mato Grosso (Oliveira-Filho and Martins 1991) in the Central-West region of Brazil, where the species-rich (Ratter et al. 1997) “Cerrado” (tropical savannah) vegetation is dominant (Oliveira-Filho and Martins 1991) and waterfalls, caves, and ponds are common. Finally, *Selaginella sematophylla* seems to be the most widely distributed species of all the spike mosses newly described herein, as it is recorded from Campos Rupestres vegetation in the localities of São Sebastião do Paraíso and Parque Estadual de Serra Nova, part of the Espinhaço Mountain Range, in the state of Minas Gerais and in mountane areas with some remnants of Atlantic Rainforest vegetation such as Pedra do Garrafão in Santa Maria do Jetibá, state of Espírito Santo and Santo Antônio do Imbé in the state of Rio de Janeiro. Because of their restricted currently documented distributions to one or two localities within a single Brazilian state, six of these new species, except for *Selaginella sematophylla*, are tentatively considered local endemics.

Additionally, we propose the following updates to Hirai’s (2015) list: *Selaginella arenaria* Baker = *Selaginella brevifolia* Baker (Valdespino 2015), Selaginella bahiensis subsp. manausensis (Bautista) Jermy & Rankin (≡ *Selaginella manausensis* Bautista) is conspecific with and a synonym of *Selaginella palmiformis* Alston ex Crabbe & Jermy (which see for details), *Selaginella cladorrhizans* A. Braun = *Selaginella tenella* (P. Beauv.) Spring (Valdespino 1995, Mickel et al. 2004), *Selaginella fragillima* Silveira is conspecific with and a synonym of *Selaginella vestiens* Baker (see discussion under *Selaginella sematophylla* and *Selaginella vestiens*), and *Selaginella pedata* Klotzch = *Selaginella parkeri* (Hook. & Grev.) Spring (Alston et al. 1981). Furthermore, *Selaginella gynostachya* Valdespino and *Selaginella sandwithii* Alston, reported from Brazil by Góes-Neto et al. (2015) should be added to Hirai’s account as well. Accordingly, there are 58 well-documented native Brazilian *Selaginella* species and if we were to take into account the introduced taxa listed by Hirai (2015), i.e., *Selaginella kraussiana* (Kunze) A. Braun [native of Africa and Macaronesia (Alston et al. 1981)], *Selaginella plana* (Desv. ex Poir.) Hieron. [native of Southeast Asia and Indonesia (Valdespino 1993b), and *Selaginella vogelii* Spring [native of Africa (Stefanović et al. 1997)], then a total of 61 species of *Selaginella* would be recorded for Brazil. These statistics are likely to change as work on Brazilian *Selaginella* continues.

## Material and methods

Herbarium specimens were examined from B, BHCB, BM, CAS, CESJ, COL, G, GH, INPA, K, MG, MO, NY, P, PMA, QCA, R, RB, UC, US, and W (Thiers 2015) and samples for Scanning Electron Microscopy (SEM) were taken from selected collections to document upper and lower surfaces of stems and leaves, as well as spore morphology. Although for each of the new species an effort was made to secure megaspore and microspore samples to determine sculpturing pattern, color, and diameter, these were not always available or, in some cases, were too immature to be utilized for those purposes. The SEM samples were prepared, viewed, and photographed at different magnifications using a Zeiss Model Evo 40 at 20–30 KV following standard techniques as described by Valdespino (1995) and Valdespino et al. (2014). Digitized SEM images were post-processed with Adobe Photoshop and assembled according to species in multipart figures.

In heterophyllous species of *Selaginella* (i.e., subg. *Stachygynandrum*, where the new taxa are classified, and subg. *Heterostachys* Baker) there are three kinds of vegetative leaves (i.e., lateral/ventral, median/dorsal, and axillary). The axillary leaves are located ventrally at branch forks on dorsiventral shoots and are usually morphologically similar to lateral leaves (Schoute 1938, Valdespino 1995) and, thus, in previous descriptions vegetative leaves are often referred to as “dimorphic”. Nevertheless, on occasion, axillary leaves may be quite different morphologically from lateral leaves (Valdespino 1995) and to take this into account we decided to use the term “heteromorphic” when describing vegetative leaves in our species descriptions. Likewise, sporophylls are described as “monomorphic” because no significant differences in their size and form were found; however, their epidermal cell composition may be different according to their plane of insertion on the strobilus axis with respect to the main stem, which allows two types to be recognized: “dorsal sporophylls” (inserted in the same plane as the median/dorsal leaves) and “ventral sporophylls” (inserted in the same plane as the lateral/ventral leaves). Otherwise, descriptions of the new species were made according to terminology utilized by Valdespino (1995), while leaf and spore measurement methods and the terminology used to describe leaf surfaces are those explained in Valdespino et al. (2014). The description of spore morphology follows Valdespino (1995), Punt et al. (2007), and Hesse et al. (2009).

## Taxonomy

### Selaginella alstonii

Taxon classificationPlantaeSelaginellalesSelaginellaceae

G. Heringer, Salino & Valdespino sp. nov.

urn:lsid:ipni.org:names:77147598-1

Figures 1 , 2

#### Diagnosis.

*Selaginella alstonii* resembles *Selaginella acanthostachys* Baker, from which it differs by having the upper surfaces of the lateral leaves glabrous (vs. hairy near basiscopic margins), median leaves acuminate to short-aristate (vs. long-aristate) with each acumen (arista) ¼ or less the lamina length (vs. arista ⅓–½), with the outer and inner hyaline margins about the same width (vs. outer margin almost twice as wide as the inner one), and non stoloniferous stems (vs. stoloniferous).

#### Type.

**BRAZIL**. Minas Gerais: Santo Antônio do Itambé, Parque Estadual do Pico do Itambé, 18°23'50,4"S, 43°19'55,5"W, 1676 m, 5 Oct 2006, *T.E. Almeida et al. 533* (holotype: BHCB!; isotype: PMA!).

#### Description.

*Plants* epipetric. *Stems* prostrate to ascending, greenish to stramineous, to 10 cm long, 0.3–0.6 mm diam., exarticulate, not flagelliform or stoloniferous, 2- or 3-branched. *Rhizophores* ventral, borne on the proximal ⅔ of stems, filiform, 0.1–0.2 mm diam. *Leaves* heteromorphic throughout, chartaceous, both surfaces glabrous, upper surfaces green, lower surfaces silvery green. *Lateral leaves* distant, spreading to slightly ascending, oblong to oblong-lanceolate, 1.1–2.0 × 0.4–1.0 mm; bases rounded, acroscopic bases overlapping stems, basiscopic bases free from stems; acroscopic margins on upper surfaces hyaline along proximal ½–¾ in a band 1 or 2 cells wide, the cells elongate and papillate parallel to margins, papillae in 1 row over each cell lumen, otherwise greenish distally with rounded to quadrangular, sinuate-walled cells, on lower surfaces hyaline in a band 2–5 cells wide, the cells elongate and papillate parallel to margins, papillae in 1 row over each cell lumen, short-ciliate along proximal ⅓–½, otherwise serrate distally, basiscopic margins greenish on upper surfaces with rounded to quadrangular, sinuate-walled cells and on lower surfaces with elongate, sinuate-walled cells, entire along proximal ¾ and serrulate on distal ¼; apices acute to slightly cuspidate, each cusp 0.02–0.03 mm, tipped by 1–3 teeth; upper surfaces comprising rounded to quadrangular, sinuate-walled cells, without idioblasts or stomata, lower surfaces comprising elongate, sinuate-walled cells, with some obscure, papillate idioblasts and stomata along central portion of midribs and along basiscopic margins. *Median leaves* distant to slightly imbricate near the branch tips, ascending, elliptic to elliptic-lanceolate or ovate-elliptic, 0.7–1.4 × 0.4–0.7 mm; bases oblique; margins hyaline in a band 2–5 cells wide, the cells elongate and papillate parallel to margins, papillae in 1 row over each cell lumen, inner margins serrate to short-ciliate, outer margins entire along proximal ½, otherwise serrate to short-ciliate distally; apices acuminate to short-aristate, each acumen (arista) 0.15–0.2 mm, entire or obscurely tipped by 1–3 teeth; both surfaces without conspicuous idioblasts, upper surfaces comprising quadrangular to rounded, sinuate-walled cells, some of these covered by 10–20 papillae, with stomata along midribs on distal half and submarginal and marginal along proximal half of outer margins, lower surfaces comprising elongate, sinuate-walled cells, without stomata. *Axillary leaves* similar to lateral leaves but with both margins ciliate along proximal ¼, otherwise short-ciliate to serrate distally. *Strobili* terminal on branch tips, compact, quadrangular, 1.5–4.0 mm. *Sporophylls* monomorphic, without a laminar flap, ovate to ovate-lanceolate, 0.7–1.1 × 0.4–0.6 mm, each with a dentate (teeth often caducous) keel along distal ½ of the midribs; bases rounded; margins narrowly hyaline, serrate; apices acute, entire or obscurely tipped by 1–3 teeth; *dorsal sporophylls* with upper surfaces green and cells as in median leaves, except for the half that overlaps the ventral sporophylls, there hyaline with elongate, sinuate-walled cells, lower surfaces silvery green and comprising elongate, sinuate-walled cells; *ventral sporophylls* with both surfaces hyaline to faintly greenish hyaline, comprising elongate, sinuate-walled cells. *Megasporangia* in proximal portion in 2 ventral rows; *megaspores* cream, with a cristate equatorial flange, rugulate on proximal faces, reticulate with low, cristate ridges on distal faces, with areolate-perforate microstructure on both faces, 250–300 μm diam. *Microsporangia* in 2 dorsal rows and, in distal portion, also in 2 ventral rows; *microspores* orange, psilate marginally and verrucate-rugulate towards the center with psilate microstructure on proximal faces, clavate (Fig. 2G, H) or echinulate to baculate (if apices of projected elements broken off, Fig. 2F, H) with striate to striate-reticulate microstructure on distal faces, 27–33 μm diam.

#### Habitat and distribution.

*Selaginella alstonii* is epipetric on rocky caves in Campos Rupestres vegetation; the type and paratype were collected at an elevation range of 1676–1810 m. The species is known only from Parque Estadual do Pico do Itambé in Serra do Espinhaço, Minas Gerais, Brazil, where it may be a local endemic.

#### Etymology.

*Selaginella alstonii* is named for Arthur Hugh Garfit Alston (1902–1958), a British pteridologist and one of the world’s authorities on the genus *Selaginella*.

#### Conservation status.

There is limited information on the conservation status and range distribution of *Selaginella alstonii*. Nevertheless, given that the localities where this species is presently known are located within the Espinhaço Mountain Range, a habitat threatened by human activities (Rapini et al 2008), we tentatively consider it vulnerable (VU) according to IUCN (2012) categories and criteria.

#### Additional specimen examined (paratype).

**BRAZIL**. **Minas Gerais**: Santo Antônio do Itambé, Parque Estadual do Pico do Itambé, 18°23'50,4"S, 43°19'55,5"W, 1810 m, 5 Oct 2006, *Almeida et al. 535* (BHCB).

#### Discussion.

**Figure 1.**
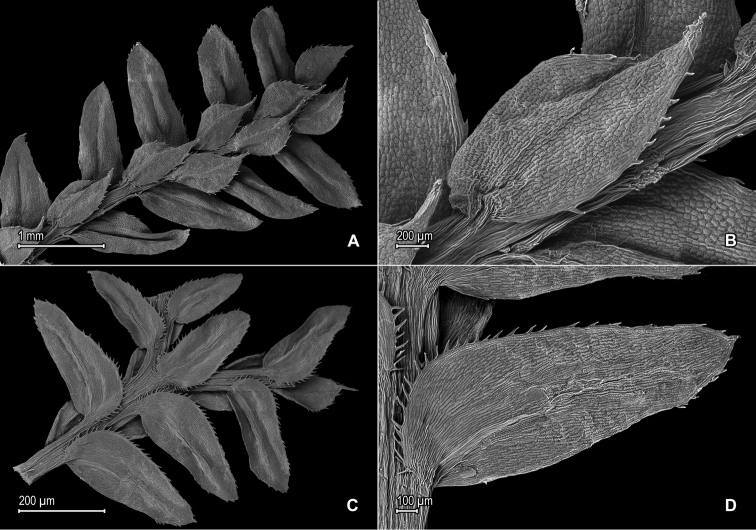
*Selaginella alstonii* G. Heringer, Salino & Valdespino. **A** Section of upper surface of stem **B** Upper surface of median leaf **C** Section of lower surface of stem **D** Lower surface of lateral leaf. **A–D** taken from isotype, *Almeida et al. 533* (PMA).

*Selaginella alstonii* belongs to subg. *Stachygynandrum* and is characterized by its oblong to oblong-lanceolate lateral leaves with acroscopic margins short-ciliate along proximal ⅓–½ and elliptic to elliptic-lanceolate or ovate-elliptic median leaves with oblique bases (Fig. 1). Dried specimens of *Selaginella alstonii* tend to develop a groove along midribs of lateral leaves (Fig. 1A), but it remains to be confirmed if this is a character observed in living plants or an artifact when plants are dried. The surfaces of the median and lateral leaves of *Selaginella alstonii* do not show conspicuous idioblasts when observed with a stereoscope, but on SEM micrographs, idioblast-like, papillate elongate cells are observed on the lower surfaces of lateral leaves, with papillae in 1 row over each cell lumen, parallel to the midribs (Fig. 1D). Additionally, in some median leaves, the outer bases have 2–4 short cilia. In some plants of *Selaginella alstonii*, as well as in *Selaginella saltuicola* (which see for discussion), we observed vegetative growth from the tips of some strobili.

*Selaginella alstonii* resembles *Selaginella acanthostachys* from Colombia, Ecuador, and Peru; however the characters given in the diagnosis separate them. Among other species of *Selaginella* from Minas Gerais, *Selaginella alstonii* may be confused with *Selaginella decomposita* Spring because of their similar texture and shape of the lateral leaves. *Selaginella decomposita*, however, has an ascending to erect habit and is a more robust plant.

**Figure 2.**
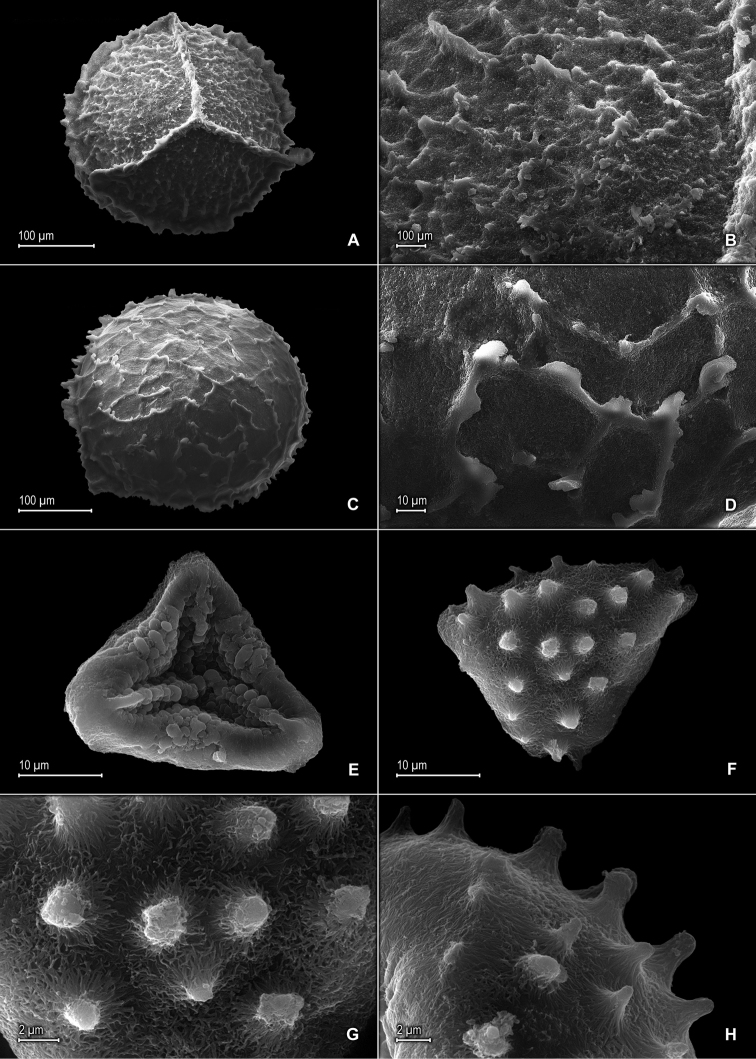
*Selaginella alstonii* G. Heringer, Salino & Valdespino. **A** Megaspore proximal face **B** Close-up of megaspore proximal face surface **C** Megaspore distal face **D** Close-up of megaspore distal face surface **E** Microspore proximal face **F** Microspore distal face **G** Close-up of microspore distal face surface **H** Close-up of microspore equatorial view surface **A–H** taken from isotype, *Almeida et al. 533* (PMA).

### Selaginella blepharodella

Taxon classificationPlantaeSelaginellalesSelaginellaceae

Valdespino sp. nov.

urn:lsid:ipni.org:names:77147599-1

Figures 3 , 4 , 5

#### Diagnosis.

*Selaginella blepharodella* differs from similar *Selaginella thysanophylla* A.R. Sm. in its short- (vs. non-) stoloniferous stems, median leaves broadly-ovate to ovate-elliptic (vs. ovate or orbicular), 0.6–1.0 × 0.4–0.6 (vs. 1.4 × 1.0) mm, with stomata on upper surfaces restricted to the midribs (vs. also on submarginal and marginal regions on proximal ¼ near outer bases), lateral leaves broadly ovate to ovate-oblong (vs. ovate or orbicular), 0.8–2.0 × 0.5–0.8 (vs. 2.0 × 1.5) mm, and upper surfaces of sporophylls with long or short cilia along distal ½ of the midribs (vs. upper surfaces glabrous).

#### Type.

**BRAZIL**. Bahia: Ibicoara, [13°24'00"S, 41°18'00"W], 26 Aug 2009, *P. Moraes & van der Werff 2933* (holotype: PMA!; isotypes: HUEFS-n.v., MO!, UC!).

#### Description.

*Plants* terrestrial or epipetric. *Stems* decumbent to suberect, stramineous, 1.5–9.5 cm long, 0.3–0.4 mm diam., exarticulate, not flagelliform, short-stoloniferous, 1- or 2-branched. *Rhizophores* axillary or axillary-ventral, restricted to bases of stems, filiform, 0.1–0.2 mm diam. *Leaves* heteromorphic throughout, chartaceous, those on and above first branch of stems with both surfaces usually glabrous and those below the first branch of stems often with few, caducous cilia-like or dentate projections on the upper surfaces of the median leaves and sporophylls and on the lower surfaces of lateral leaves, upper surfaces green or brownish (when old), lower surfaces silvery green or shiny brown (when old). *Lateral leaves* imbricate, spreading or ascending, broadly ovate to ovate-oblong, 0.8–2.0 × 0.5–0.8 mm; bases rounded to subcordate, acroscopic bases overlapping stems (more so on leaves below first branch), basiscopic bases free from stems; acroscopic margins broadly hyaline, especially along proximal ⅓ in a band 3–15 cells wide, the cells elongate and papillate parallel to margins, papillae in 1 or 2 rows over each cell lumen, long-ciliate along proximal ⅔ and short-ciliate to serrate along distal ⅓, basiscopic margins hyaline to greenish hyaline in a band 4–6 cells wide, the cells as on acroscopic margins, ciliate throughout or sometimes cilia absent from proximal ¼–⅓; apices acute, tipped by 1–4 teeth or 2 or 3 cilia, especially on leaves below first branch; upper surfaces comprising quadrangular to rounded, sinuate-walled cells covered by 5–15 papillae, without idioblasts or stomata, lower surfaces comprising elongate, sinuate-walled cells, most of these papillate and idioblast-like, papillae in 1 or 2(–3) rows over each cell lumen, with stomata in 2 or 3(–4) rows along midribs and some along proximal ¼ of basiscopic margins. *Median leaves* imbricate, ascending, broadly-ovate to ovate-elliptic, 0.6–1.0 × 0.4–0.6 mm; bases oblique, inner bases truncate, outer bases rounded and glabrous or these may also be ventricose (i.e., swollen) and each with a tuft of long cilia on leaves below first branch; margins broadly hyaline, especially the inner ones, in a band 5–15 cells wide, the cells elongate and papillate parallel to margins, papillae in 1 or 2 rows over each cell lumen, long-ciliate throughout or infrequently along only distal ⅘; apices gradually tapering into a long acumen, each acumen 0.1–0.3 mm, tipped by 2 or 3 cilia; both surfaces without idioblasts, upper surfaces comprising quadrangular to rounded, sinuate-walled cells covered by 5–15 papillae, with stomata along midribs, lower surfaces comprising elongate, sinuate-walled cells, without stomata. *Axillary leaves* similar to lateral leaves. *Strobili* terminal on branch tips, compact, quadrangular, 1.2–9.0 mm long. *Sporophylls* monomorphic, without a laminar flap, ovate, 0.6–0.9 × 0.4–0.7 mm, each with a ciliate keel along distal ½ of the midribs; bases rounded; margins hyaline, long-ciliate; apices acute, tipped by 1 or 2 cilia; *dorsal sporophylls* with upper surfaces green and cells as in median leaves, except for the half that overlaps the ventral sporophylls, there hyaline with elongate, papillate, and slightly sinuate-walled cells, lower surfaces silvery green and comprising elongate, sinuate-walled cells (Fig. 4F); *ventral sporophylls* with both surfaces hyaline to greenish, comprising elongate, sinuate-walled cells. *Megasporangia* in proximal portion in 2 ventral rows; *megaspores* light-yellow, rugulate-reticulate on proximal faces, reticulate on distal faces, with psilate-perforate microstructure on both faces, 200–230 μm diam. *Microsporangia* in 2 dorsal rows and, in distal portion, also in 2 ventral rows; *microspores* orange, verrucate-rugulate with granulate microstructure on proximal faces, broadly capitate to clavate (5B–D) or broadly baculate (if apices of projected elements broken off, Fig. 5G, H) with reticulate-perforate and echinulate microstructure on distal faces, ca. 30–38 μm diam.

#### Habitat and distribution.

*Selaginella blepharodella* is presumed to be a local endemic of the Serra do Sincorá, Espinhaço Range, state of Bahia, Brazil, where it is known from only two localities, growing on sandy soil or overhanging from rocks at 1400 m.

#### Etymology.

The epithet of the new species derives from the Greek *blepharis*, meaning eyelash, *ode* meaning similar to and *ella*, Latin diminutive suffix; this refers to the long-ciliate leaf margins that resemble miniature eyelashes.

#### Conservation status.

*Selaginella blepharodella* is known from only two collections in Serra do Sincorá and may be expected to occur in places with similar vegetation types in the Chapada Diamantina region of the Espinhaço Mountain Range. The Chapada Diamantina region and the Espinhaço Mountain Range, in general, are still subject to anthropomorphic pressure, including low-scale mining (Pedreira 2002), subsistence agriculture accompanied by the slash-and-burn methods, and plant extraction for commerce (Rapini et al. 2008). Based on these threats and according to IUCN (2012) categories and criteria, this species is tentatively considered vulnerable (VU).

#### Additional specimen examined (paratype).

**BRAZIL**. **Bahia**: Serra do Sincorá, 1400 m, Nov 1906, *Ule 7298* (B, BM, PMA-fragment).

#### Discussion.

**Figure 3.**
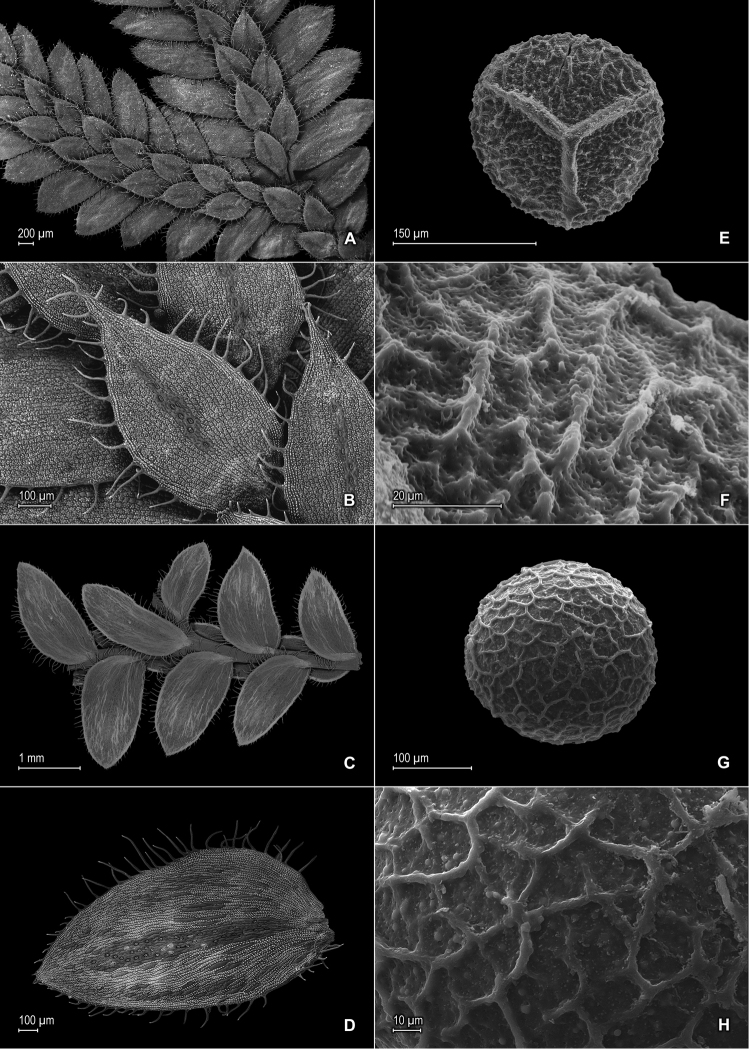
*Selaginella blepharodella* Valdespino. **A** Section of upper surface of stem **B** Upper surface of median leaf **C** Section of lower surface of stem **D** Lower surface of lateral leaf **E** Megaspore proximal face **F** Close-up of megaspore proximal face surface **G** Megaspore distal face **H** Close-up of megaspore distal face surface **A–D** taken from holotype, *Moraes & van der Werff 2933* (PMA) **E–H** taken from paratype, *Ule 7298* (B).

**Figure 4.**
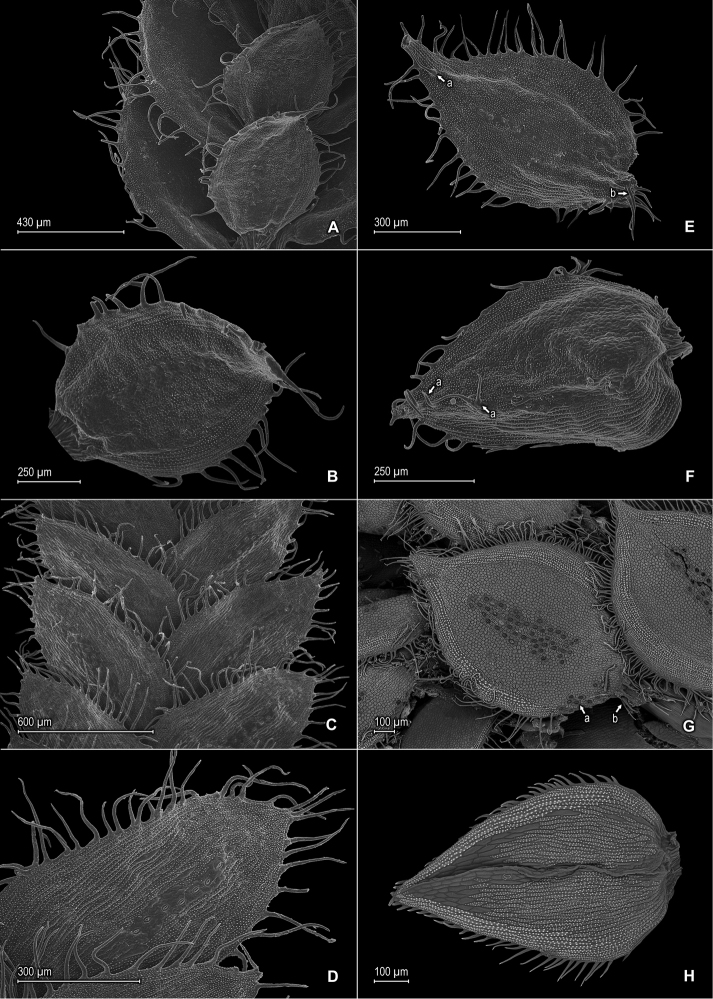
*Selaginella blepharodella* Valdespino. **A** Section of upper surface of stem **B** Upper surface of median leaf **C** Section of lower surface of stem **D** Lower surface of lateral leaf **E** Upper surface of median leaf, note: cilium-like or tooth projection (a) and long cilia on outer base (b) **F** Upper surface of dorsal sporophyll, note: cilia-like or teeth projections on distal portion of midrib (a) **A–F** taken from paratype *Ule 7298* (B). *Selaginella thysanophylla* A.R. Sm. **G** Upper surface of median leaf, note: stomata on submarginal and marginal regions on proximal ¼ near outer base (a) and outer base tufted with long cilia (b) **H** Upper surface of ventral sporophyll **G, H** taken from *Steyermark et al. 113322* (NY).

*Selaginella blepharodella* is a member of subg. *Stachygynandrum* and is defined here in a broad sense to encompass the morphological variability found within the two collections examined. In general, this species is characterized by long-ciliate leaves with broadly hyaline margins, lateral leaves imbricate, spreading to ascending with lower surfaces almost completely comprising elongate, papillate, sinuate-walled cells with papillae in 1–3 rows over cell lumina and stomata in 2 or 3(–4) rows along midribs amidst shortly elongate, sinuate-walled cells, and median leaves with apices ending in 2 or 3 cilia (Figs 3, 4). The type collection (*Moraes & van der Werff 2933*) has stems more than 3 cm tall, is 2- or 3-brached, and has lateral leaves mostly spreading to ascending and imbricate at branch tips (Fig. 3), whereas the paratype (*Ule 7298*) is a much smaller plant to 3 cm tall, is 1- or 2-branched, and has lateral leaves imbricate throughout (Fig. 4). In both specimens, the leaves below the first branch tend to be more imbricate, have wider hyaline margins and longer marginal cilia, and the outer bases of the median leaves may be ventricose and with a tuft of long cilia (Fig. 4E). Additionally, below the first branch, they may have scarce and caducous cilia-like projections on the upper surfaces of the median leaves and sporophylls (Fig. 4E, F) and on the lower surfaces of lateral leaves. In these characters, *Selaginella blepharodella*, especially the paratype, is similar and perhaps related to *Selaginella thysanophylla* from Venezuela (Fig. 4G). These two species also share similar megaspore color; however *Selaginella blepharodella* can be separated from *Selaginella thysanophylla* by the characters discussed in the diagnosis. *Selaginella blepharodella* differs further from *Selaginella thysanophylla* by having megaspores 200–230 (vs. 150–200) μm, lateral leaves with acute (vs. rounded to subacute) apices, median leaves with the inner margins hyaline in a band 5–15 (vs. 20–25) cells wide at least along proximal ⅓ with long-acuminate (vs. apiculate) apices, each acumen 0.1–0.3 (vs. acumen 0.05–0.1) mm, and sporophyll apices each tipped by 2 cilia (Fig. 4F) [vs. 2 teeth; (Fig. 4H)].

In Brazil, *Selaginella blepharodella* does not seem to have close relatives, but it shares some characters, e.g., hyaline and ciliate leaf margins, with the newly described *Selaginella mucugensis* and *Selaginella crinita* (which see for comparison).

**Figure 5.**
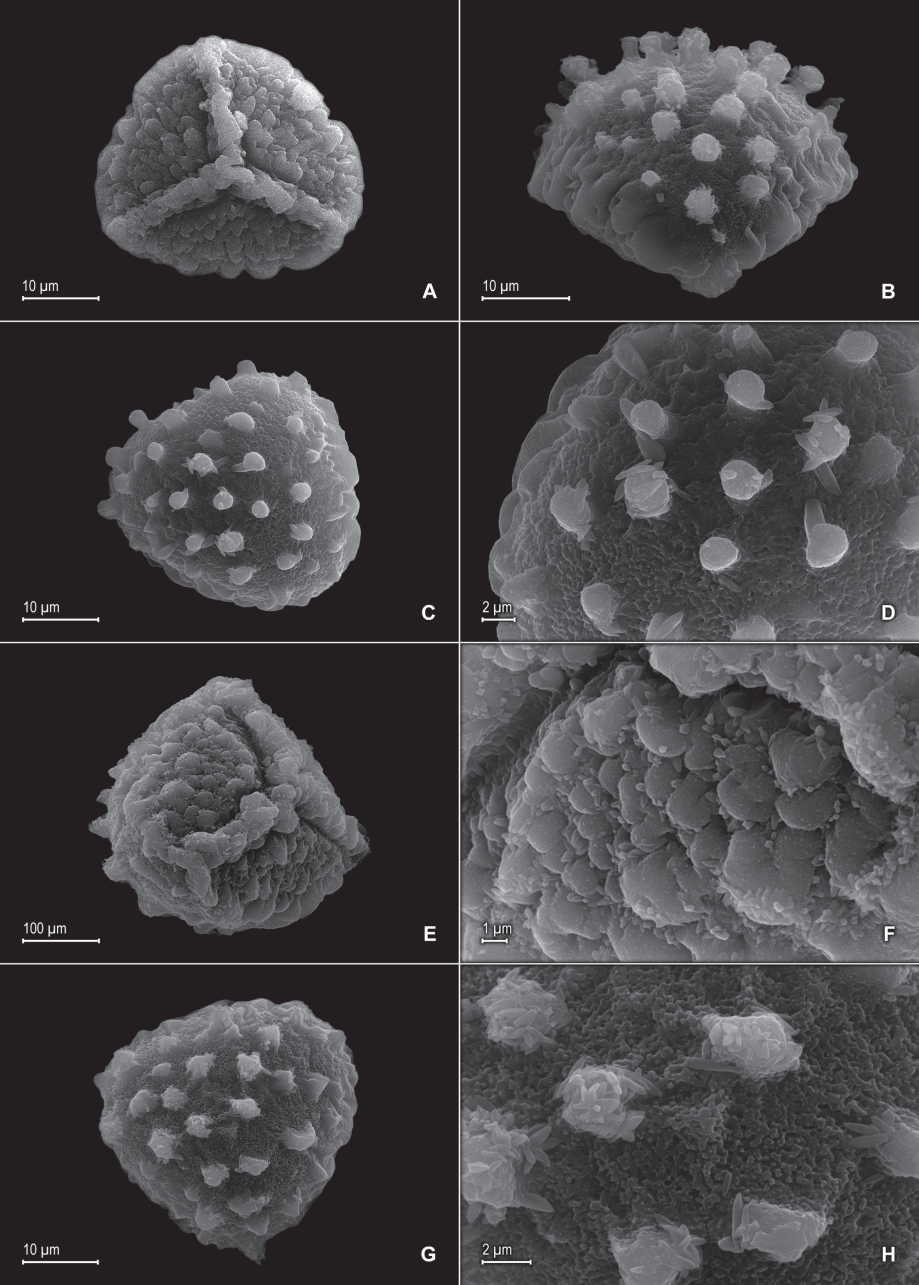
*Selaginella blepharodella* Valdespino. **A** Microspore proximal face **B** Microspore equatorial view **C** Microspore distal face **D** Close-up of microspore distal face surface **E** Microspore proximal-equatorial view **F** Close-up of microspore proximal face surface **G** Microspore distal face **H** Close-up of microspore distal face surface **A–H** taken from holotype, *Moraes & van der Werff 2933* (PMA).

### Selaginella crinita

Taxon classificationPlantaeSelaginellalesSelaginellaceae

Valdespino sp. nov.

urn:lsid:ipni.org:names:77147600-1

Figures 6 , 7

#### Diagnosis.

*Selaginella crinita* is morphologically similar to and may be confused with the Brazilian endemic, *Selaginella jungermannioides* (Gaudich.) Spring, but differs in its lateral leaves long-ciliate throughout the basiscopic margins (vs. along proximal ¼ and then serrulate distally), median leaves with margins long-ciliate throughout (vs. along proximal ¼, particularly on outer margins, otherwise short-ciliate to serrulate distally), and apices long-acuminate (vs. cuspidate to acuminate) with each acumen hyaline (vs. cusp or acumen green) tipped by 2–4 long cilia (vs. entire).

#### Type.

**BRAZIL**. Bahia: Mun. Água Quente, Pico das Almas, Vertente Oeste, trilho do povoado da Sta. Rosa, 35 km W of the city, 13°31'S, 42°00'W, 1100–1300 m, 1 Dec 1988, *R. Harley & N. Taylor 27048* (holotype: NY!; isotypes: BM-n.v., CEPEC-n.v., K-n.v., PMA!, SPF-n.v.).

#### Description.

*Plants* terrestrial. *Stems* prostrate, stramineous, to 10 cm long, 0.3–0.5 mm diam., exarticulate, not flagelliform or stoloniferous, 1- or 2-branched. *Rhizophores* axillary, borne throughout stems, filiform, 0.1–0.2 mm diam. *Leaves* heteromorphic throughout, chartaceous, both surfaces glabrous, upper surfaces green or brownish (when old), lower surfaces silvery green or shiny brown (when old). *Lateral leaves* imbricate or distant, spreading to ascending, ovate-oblong, 1.5–2.0 × 0.5–1.0 mm; bases rounded, acroscopic bases strongly overlapping stems, basiscopic bases free from stems; acroscopic margins hyaline in a band 2–7 cells wide, the cells elongate and papillate parallel to margins, papillae in 1 or 2 rows over each cell lumen, long-ciliate along proximal ⅔ and short-ciliate along distal ⅓; basiscopic margins greenish to slightly hyaline in a band 1 or 2 cells wide, the cells as along acroscopic margins, long-ciliate throughout, apices obtuse to rounded, variously tipped by 1–5 cilia; upper surfaces comprising quadrangular to rounded, sinuate-walled cells, most of these covered by 15–30 papillae, without idioblasts or stomata, lower surfaces comprising elongate, sinuate-walled cells, most of these papillate and idioblast-like, papillae in 1 or 2 rows over each cell lumen, with stomata in 1 or 2 rows along midribs where cells are shortly elongate and sinuate. *Median leaves* imbricate, ascending, ovate-lanceolate to ovate-elliptic, 1.0–1.5 × 0.4–0.7 mm; bases rounded to truncate; margins hyaline in a band 2–5 cells wide, the cells elongate and papillate parallel to margins, papillae in 1 or 2 rows over each cell lumen, long-ciliate throughout; apices gradually tapering into a long-acumen, each acumen 0.12–0.15 mm, tipped by 2–5 cilia; both surfaces without idioblasts, upper surfaces comprising quadrangular to rounded, sinuate-walled cells covered by 15–30 papillae, with stomata along midribs, lower surfaces comprising elongate, sinuate-walled cells, without stomata. *Axillary leaves* ovate-oblong to oblong, otherwise similar to lateral leaves. *Strobili* terminal on branch tips, compact, quadrangular, 1.5–2.0 mm. *Sporophylls* monomorphic, without a laminar flap, ovate, 0.7–1.1 × 0.4–0.6 mm, each usually with a slightly developed and ciliate (cilia often caducous) keel along distal ½ of midribs; bases rounded; margins narrowly hyaline, long-ciliate; apices acute, tipped by 1 or 2 cilia; *dorsal sporophylls* with upper surfaces green and cells as in median leaves, except for the half that overlaps the ventral sporophylls, there hyaline with elongate, papillate, and slightly sinuate-walled cells, lower surfaces silvery green and comprising elongate, sinuate-walled cells; *ventral sporophylls* with both surfaces hyaline to greenish, comprising elongate, sinuate-walled cells. *Megasporangia* in proximal portion in 2 ventral rows; *megaspores* white to creamy, rugulate-reticulate on proximal faces, reticulate-granular on distal faces, with granulate-echinulate and perforate microstructure on both faces, 250–258 μm diam. *Microsporangia* in 2 dorsal rows and, in distal portion, also in 2 ventral rows; *microspores* orange, rugulate-verrucate on proximal faces, broadly clavate or broadly baculate (if apices of projected, echinulate elements broken off) [Fig. 7F] on distal faces, with echinulate microstructure on both faces, 25–33 µm.

#### Habitat and distribution.

*Selaginella crinita* is known only from the type collection from Pico das Almas, Serra do Rio de Contas, Bahia, Brazil, where it is probably a local endemic. It grows on shady rocky and sandy soil at 1100–1300 m.

#### Etymology.

The specific epithet is derived from the Latin *crinitus*, meaning long haired; this refers to the many, long cilia along leaf margins.

#### Conservation status.

There is insufficient data to definitively ascertain distributional range, abundance, and possible threats to this species. Nevertheless, since its type locality is in the Chapada Diamantina region of the Espinhaço Mountain Range, which is threatened by anthropomorphic activities (Rapini et al. 2008), *Selaginella crinita* is tentatively considered vulnerable (VU), according to IUCN (2012) categories and criteria.

#### Discussion.

*Selaginella crinita* is a prostrate species that belongs to subg. *Stachygynandrum* and is characterized by its median leaves ovate-lanceolate to ovate-elliptic, with the inner and the outer margins symmetric, and apices tapering into a long-acumen with each acumen tipped by 2–5 cilia, lateral leaves ovate-elliptic to ovate-oblong, as well as long-ciliate leaf margins, and quadrangular to rounded sinuate-walled cells on upper surfaces of median and lateral leaves covered by many (15–30) papillae. Because of its imbricate and long-ciliate lateral leaves and leaves tipped by cilia, *Selaginella crinita* may be confused with *Selaginella blepharodella*. *Selaginella crinita* is easily separated from *Selaginella blepharodella* by its prostrate (vs. decumbent to suberect) habit, median leaves margins hyaline in a band 2–5 (vs. 5–15) cells wide, lateral leaves with obtuse to rounded (vs. acute) apices, and the cells of upper surfaces of median and lateral leaves covered by 15–30 (vs. 5–15) papillae.

*Selaginella crinita* belongs to a Neotropical group of *Selaginella* species, here informally termed the “*Selaginella jungermannioides* group”, characterized mostly by creeping or prostrate habit, rhizophores usually distributed throughout the stems, median leaf apices acute, cuspidate, acuminate, or aristate, and lateral leaves often ovate-oblong or oblong with truncate, obtuse to broadly acute apices. The “*Selaginella jungermannioides* group” tentatively includes the South American taxa *Selaginella applanata* A. Braun (Colombia, Venezuela, and Peru), *Selaginella homaliae* A. Braun (Colombia and Brazil), *Selaginella jungermannioides* (Brazil), *Selaginella schultesii* Alston ex Crabbe & Jermy (Colombia), and *Selaginella truncata* H. Karst. ex A. Braun (Colombia, Peru, and Bolivia), as well as *Selaginella lindenii* Spring from southern Mexico. Among species in the “*Selaginella jungermannioides* group”, *Selaginella crinita* is morphologically close to *Selaginella applanata*, *Selaginella jungermannioides*, and *Selaginella lindenii*. *Selaginella crinita* can be separated from *Selaginella applanata* by its median leaves with inner and outer margins symmetric (vs. inner margins straight and outer margins convex), long-acuminate (vs. long-aristate) apices tipped by cilia (vs. entire), and acroscopic margins of lateral leaves ciliate throughout (vs. along proximal ½). It can be easily distinguished from *Selaginella lindenii* by the upper surfaces of lateral and axillary leaves glabrous (vs. hispid), whereas from *Selaginella jungermannioides* it differs by the characters of marginal projections of leaves and form of the median leaf apices, as discussed in the diagnosis. Additionally, *Selaginella crinita* may be confused with *Selaginella homaliae* and *Selaginella truncata*, but it is set apart from them by its median leaf apices long-acuminate (vs. acute to short-cuspidate) and margins long-ciliate throughout (vs. dentate to serrate in *Selaginella homaliae* and in *Selaginella truncata* inner margins denticulate and outer margins sparingly long-ciliate along proximal ⅓, otherwise denticulate). *Selaginella crinita* also differs from the newly described *Selaginella mucronata*, which may be part of the “*Selaginella jungermannioides* group”, by its median leaves ovate-lanceolate to ovate-elliptic (vs. orbiculate to broadly elliptic), with stomata on upper surfaces along midribs (vs. distributed throughout the leaf laminae), and apices long-acuminate (vs. mucronate or infrequently acute), as well as by having the cells on the upper surfaces of the lateral and median leaves covered by 15–30 (vs. 5–10) papillae.

**Figure 6.**
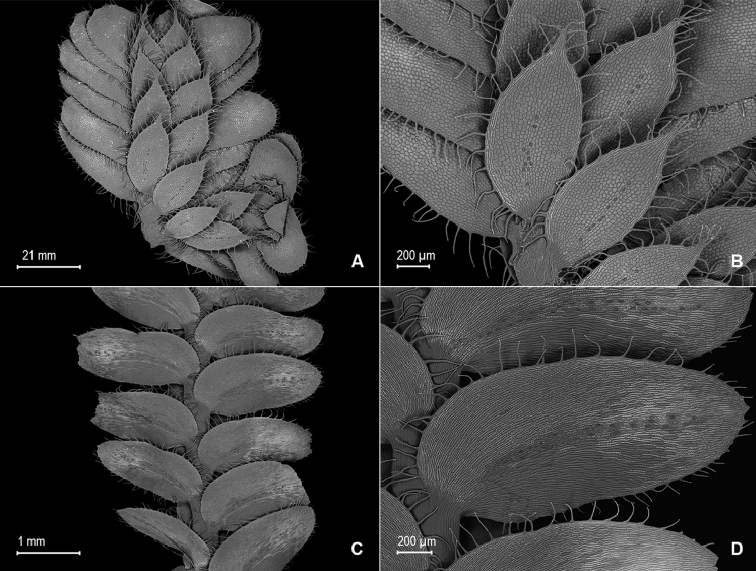
*Selaginella crinita* Valdespino. **A** Section of upper surface of stem **B** Upper surface of median leaves **C** Section of lower surface of stem **D** Lower surface of lateral leaf **A–D** taken from holotype, *Harley & Taylor 27048* (NY).

**Figure 7.**
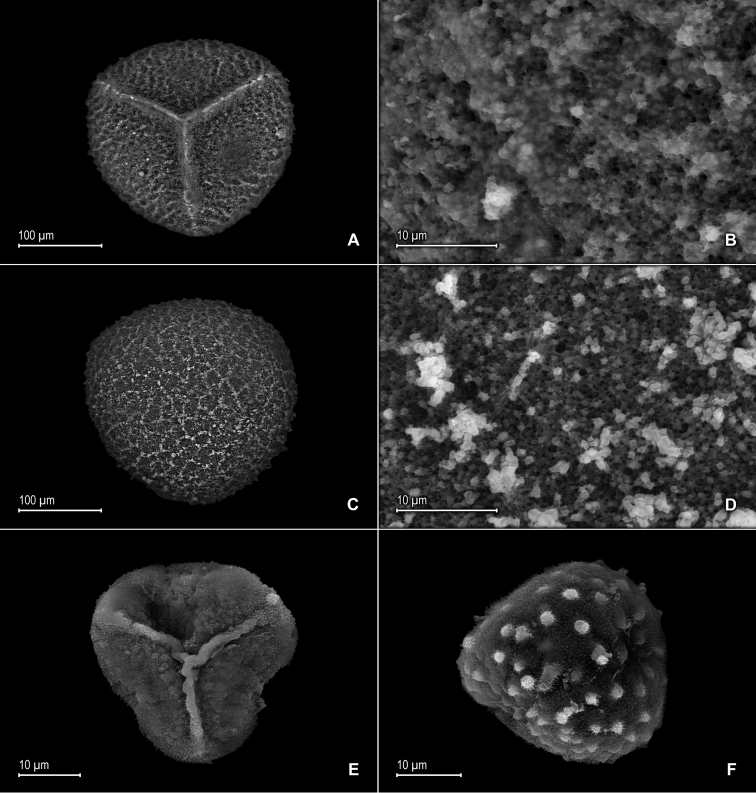
*Selaginella crinita* Valdespino. **A** Megaspore proximal face **B** Close-up of megaspore proximal face surface **C** Megaspore distal face **D** Close-up of megaspore distal face surface **E** Microspore proximal face **F** Microspore distal face **A–F** taken from holotype, *Harley & Taylor 27048* (NY).

### Selaginella mucronata

Taxon classificationPlantaeSelaginellalesSelaginellaceae

G. Heringer, Salino & Valdespino sp. nov.

urn:lsid:ipni.org:names:77147601-1

Figures 8 , 9

#### Diagnosis.

*Selaginella mucronata* seems morphologically related to *Selaginella jungermannioides* but differs from it in having the upper surfaces of the leaves slightly rugose (vs. smooth), lateral leaves with the basiscopic margins entire to serrulate distally (vs. basiscopic margins long-ciliate along proximal ⅛, otherwise entire), median leaf bases rounded (vs. oblique with a slightly developed outer lobe), and margins hyaline (vs. greenish) and long-ciliate throughout (vs. inner margins denticulate and outer margins sparingly long-ciliate along proximal ⅓, otherwise denticulate).

#### Type.

**BRAZIL**. Espírito Santo: Castelo, Parque Estadual do Forno Grande, [20°32'29"S, 41°07'17"W], [1200 m], 28 Jun 2008, *A. Salino, G. Heringer & V.A.O. Dittrich 13686* (holotype: BHCB!; isotype: PMA-fragment!).

#### Description.

*Plants* epipetric. *Stems* creeping, stramineous, 5.5–8.0 cm long, 0.4–0.8 mm diam., exarticulate, not flagelliform or stoloniferous, 1- or 2-branched. *Rhizophores* ventral, axillary or dorsal, borne throughout stems, filiform, 0.1–0.2 mm diam. *Leaves* heteromorphic throughout, membranaceous to chartaceous, both surfaces glabrous, upper surfaces green, lower surfaces silvery green. *Lateral leaves* imbricate or distant, spreading, broadly ovate to ovate-oblong, 2.5–4.0 × 1.3–2.3 mm; bases rounded or hemicordate, acroscopic bases strongly overlapping stems, basiscopic bases free from stems; acroscopic margins greenish or hyaline in band 2–4 cells wide, the cells elongate and papillate parallel to margins, papillae in 1 row over each cell lumen, long-ciliate along proximal ½–⅔ and serrulate to entire distally, basiscopic margins greenish or narrowly hyaline in a band 1 or 2 cells wide, the cells as along acroscopic margins, entire or entire along proximal ¾ and serrulate along distal ¼, apices mucronate or obtuse, tipped by 2 or 3 teeth; upper surfaces comprising rounded to quadrangular, sinuate-walled cells, most of these covered by 5–10 papillae, without idioblasts or stomata, lower surfaces comprising elongate, sinuate-walled cells, few of these papillate and idioblast-like, papillae in 1 row over each cell lumen, with stomata in 2 or 3 rows along midribs. *Median leaves* imbricate (especially at stem and branch apices), ascending, orbiculate to broadly elliptic, 1.4–2.6 × 1.1–2.1 mm; bases rounded; margins hyaline in a band 1–3 cells wide, the cells elongate and papillate parallel to margins, long-ciliate throughout; apices mucronate or infrequently acute, each mucro 0.14–0.16 mm, ending in 1–3 teeth; both surfaces without idioblasts, upper surfaces comprising rounded to quadrangular, sinuate-walled cells, many of these covered by 5–10 papillae, with stomata throughout the laminae and some near submarginal region of the outer bases, lower surfaces comprising elongate, sinuate-walled cells, without stomata. *Axillary leaves* ovate or slightly cordiform, bases rounded or cordate, margins and apices similar to lateral leaves. *Strobili* terminal on branch tips, compact, quadrangular, 4.0–7.0 mm. *Sporophylls* monomorphic to slightly dimorphic, without a laminar flap, ovate to lanceolate, 1.3–1.9 × 0.7–0.9 mm, each with or without a slightly developed denticulate keel along distal ½ of the midribs; bases rounded; margins narrowly hyaline, serrulate to short-ciliate; apices acute, tipped by 1–3 teeth; *dorsal sporophylls* with upper surfaces green and cells as in median leaves, except for the half that overlaps the ventral sporophylls, there hyaline and with elongate, papillate, and slightly sinuate-walled cells, lower surfaces silvery green and comprising elongate, sinuate-walled cells; *ventral sporophylls* with both surfaces silvery green and comprising elongate, sinuate-walled cells. *Megasporangia* in 2 ventral rows; *megaspores* creamy or light yellow, most observed immature, reticulate to reticulate-rugulate on proximal faces, reticulate on distal faces, with perforate microstructure on both faces, 200–230 µm. *Microsporangia* in 2 dorsal rows; *microspores* orange, psilate-rugulate on proximal faces, capitate or baculate (if apices of projected elements broken off) [Fig. 9E, G, H] on distal faces, with granulate microstructure on both faces, 20–27 μm diam.

#### Habitat and distribution.

*Selaginella mucronata* is known only from the type collection from Parque Estadual do Forno Grande, state of Espírito Santo, growing on rocks in understory of Atlantic Rainforest vegetation at 1200 m. It could be considered a local endemic given its limited distribution and the vegetational type.

#### Etymology.

The epithet mucronata refers to the apices of the median leaves.

#### Conservation status.

The paucity of data available does not allow an assessment of abundance and possible threats to this species and, thus, we assign to it a Data Deficient (DD) conservation assessment according to IUCN (2012) categories and criteria.

#### Discussion.

**Figure 8.**
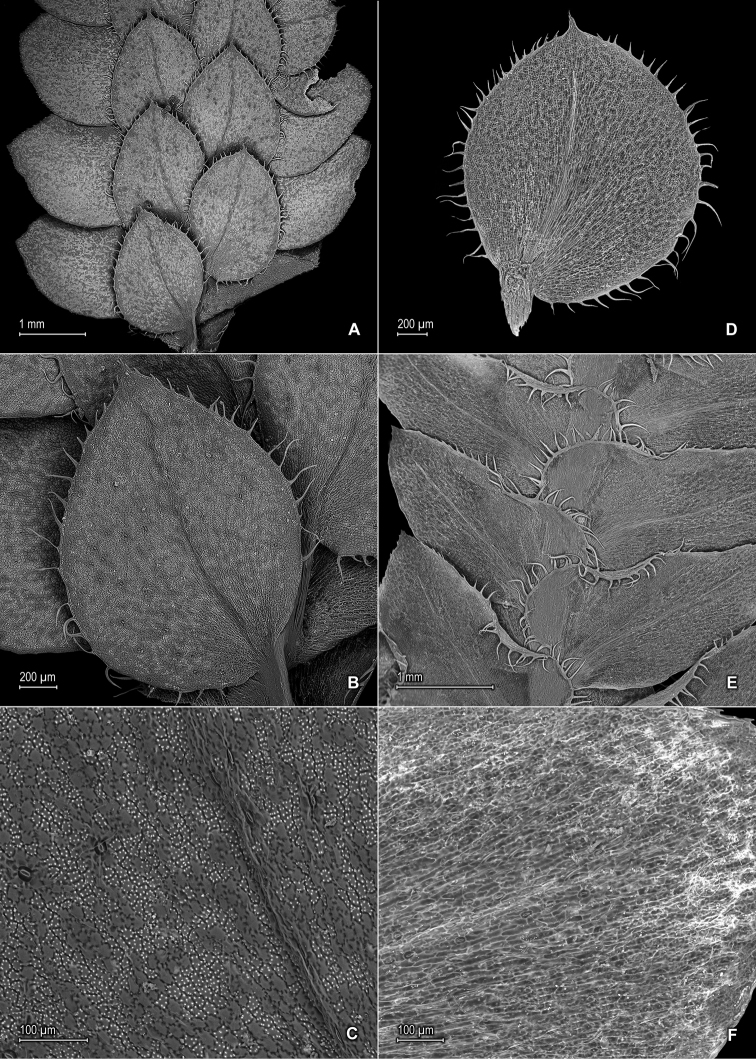
*Selaginella mucronata* G. Heringer, Salino & Valdespino. **A** Section of upper surface of stem **B** Upper surface of median leaf **C** Close-up of upper surface of median leaf **D** Lower surface of median leaf **E** Section of lower surface of stem **F** Close-up of lower surface of lateral leaf **A–F** taken from isotype, *Salino et al. 13686* (PMA).

*Selaginella mucronata* belongs to subg. *Stachygynandrum* and is characterized by its creeping habit, orbicular to broadly elliptic, long-ciliate, mucronate or infrequently acute median leaves with stomata distributed throughout the upper surfaces (Fig. 8A–C). *Selaginella mucronata* seems to be morphologically most similar to *Selaginella jungermannioides*; however, the characters of leaf texture, margin type, and shape of median leaf bases discussed in the diagnosis distinguish these two species. *Selaginella mucronata* could be confused with *Selaginella crinita*, another member of the “*Selaginella jungermannioides* group,” which see for discussion.

The upper surfaces of *Selaginella mucronata* may be slightly corrugate (Fig. 8A–C), perhaps as a drying artifact, and because of this and its creeping habit it could be confused, among other Brazilian species, with *Selaginella flexuosa* Spring and *Selaginella macrostachya* (Spring) Spring. *Selaginella mucronata* differs chiefly from those two species in having the apices of median leaves mucronate or acute (vs. long-aristate) with each acumen ^1^/_10_–^1^/_16_ (vs. arista ¼–¾) the length of the leaf lamina. Additionally, *Selaginella mucronata* differs from *Selaginella flexuosa* by acroscopic margins of lateral leaves long-ciliate along proximal ½–^2^/_3_ and serrulate to entire distally (vs. denticulate along proximal ¼–½, otherwise entire distally) and the margins of the median and axillary leaves ciliate (vs. serrulate). It is further distinguished from *Selaginella macrostachya* by its orbiculate to broadly elliptic (vs. cordate) median leaves with the outer bases glabrous (vs. tufted with short hairs) and lateral leaves with upper surfaces near basiscopic margins glabrous (vs. often with short, tooth-like hairs).

**Figure 9.**
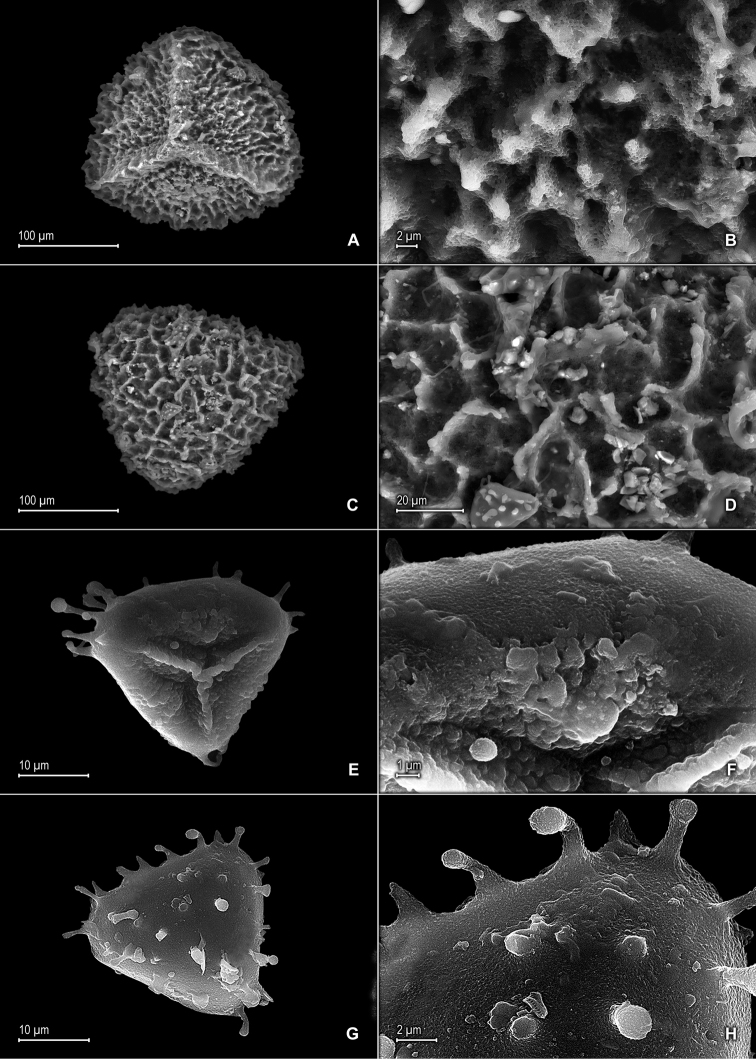
*Selaginella mucronata* G. Heringer, Salino & Valdespino. **A** Megaspore proximal face **B** Close-up of megaspore proximal face surface **C** Megaspore distal face **D** Close-up of megaspore distal face surface **E** Microspore proximal face **F** Close-up of microspore proximal face surface **G** Microspore distal face **H** Close-up of microspore distal face surface **A–H** taken from isotype, *Salino et al. 13686* (PMA).

### Selaginella mucugensis

Taxon classificationPlantaeSelaginellalesSelaginellaceae

Valdespino sp. nov.

urn:lsid:ipni.org:names:77147602-1

Figures 10 , 11

#### Diagnosis.

*Selaginella mucugensis* differs from *Selaginella blepharodella* in having median leaves distant (vs. imbricate), ovate (vs. broadly-ovate to ovate-elliptic), with margins hyaline in a band 2–5 (vs. 5–15) cells wide with cilia 30–50 (vs. 130–180) µm long, stomata on upper surfaces on submarginal and marginal regions of the outer bases (vs. restricted to midribs), apices acute to short-acuminate (vs. long-acuminate), the acumen, if present, 0.02–0.08 (vs. acumen 0.1–0.3) mm, and lateral leaves with basiscopic margins entire along proximal ¼–½ and serrate to short-ciliate distally (vs. usually ciliate throughout).

#### Type.

**BRAZIL**. Bahia: Mucugê, campo defronte ao cemitério, [ca. 13°00'S, 41°22'19"W], [ca. 984 m], 20 Jul 1981, *A.M. Giulietti et al. [CFCR 1430]* (holotype: NY!; isotypes: PMA- fragment!, SPF-n.v.).

#### Description.

*Plants* terrestrial. *Stems* ascending to suberect, stramineous, 3–7 cm long, 0.2–0.4 mm diam., exarticulate, not flagelliform, probably shortly stoloniferous, 2- or 3-branched. *Rhizophores* axillary and axillary-dorsal, restricted to the bases of stems, filiform, 0.1–0.2 mm diam. *Leaves* heteromorphic throughout, chartaceous, both surfaces glabrous, upper surfaces green, lower surfaces silvery green or brownish (when old). *Lateral leaves* distant, slightly ascending, ovate to slightly ovate-oblong, 1.2–1.5 × 0.9–1.1 mm; bases rounded, acroscopic bases slightly to strongly overlapping stems, basiscopic bases free from stems; acroscopic margins hyaline in a band 2–8 cells wide, the cells elongate and papillate parallel to margins, papillae in 1 row over each cell lumen, long- to short-ciliate along proximal ¾ and serrate to entire along distal ¼; basiscopic margins narrowly hyaline or greenish in a band 1 or 2 cells wide, the cells as along acroscopic margins, entire along proximal ¼–½ and serrate to short-ciliate distally, apices acute, tipped by 1–3 teeth; upper surfaces comprising rounded to quadrangular, sinuate-walled cells, some of these covered by 4–8 papillae, without idioblasts or stomata, lower surfaces comprising elongate, sinuate-walled cells, some of these papillate and idioblast-like, papillae in 2 rows over each cell lumen, with stomata in 2 or 3 rows along midribs and along proximal ½ of basiscopic margins. *Median leaves* distant, ascending, ovate, 0.8–1.3 × 0.5–0.7 mm; bases oblique, inner bases plane in profile, outer bases ventricose (i.e., swollen); margins hyaline in a band 2–5 cells wide, the cells elongate and papillate parallel to margins, papillae in 1 row over each cell lumen, shortly ciliate throughout; apices acute to short-acuminate, each acumen 0.02–0.08 mm, occasionally with 1 or 2 hairs on upper surfaces, tipped by 1–3 teeth; both surfaces without idioblasts, upper surfaces comprising rounded to quadrangular, sinuate-walled cells covered by 4–8 papillae, with stomata along midribs and some on submarginal and marginal regions of the outer bases, lower surfaces comprising elongate, sinuate-walled cells, without stomata. *Axillary leaves* similar to lateral leaves. *Strobili* terminal on branch tips, compact, quadrangular, 2.0–7.0 mm. *Sporophylls* monomorphic, without a laminar flap, ovate, 0.8–1 × 0.4–0.5 mm, each with a well-developed, frequently puberulous keel along the midribs; bases rounded; margins hyaline, short-ciliate to serrate; apices acute, tipped by 1 or 2 teeth; *dorsal sporophylls* with upper surfaces green and cells as in median leaves, except for the half that overlaps the ventral sporophylls, there hyaline with elongate, papillate, and slightly sinuate-walled cells, lower surfaces silvery green and comprising elongate, sinuate-walled cells; *ventral sporophylls* with both surfaces hyaline, comprising elongate, sinuate-walled cells. *Megasporangia* frequently proximal in 2 ventral rows or the proximal megasporangia abortive and a few intermixed with microsporangia; *megaspores* lemon-yellow, mostly immature or absent, proximal faces not observed, reticulate on distal faces, 275–285 µm. *Microsporangia* in 2 dorsal rows and, in distal portion, also in 2 ventral rows; *microspores* orange, gemmate-rugulate or broadly baculate-rugulate with psilate to echinulate microstructure on proximal faces, vermiculate with echinulate microstructure on distal faces, 30–40 µm.

#### Habitat and distribution.

*Selaginella mucugensis* is known only from the type collection from Mucugê, Serra do Sincorá, in the Chapada Diamantina region of the Espinhaço Mountain Range, where it is probably a local endemic. It grows terrestrially on damp soil in Campos Rupestres vegetation at ca. 984 m.

#### Etymology.

This species is named for the type locality.

#### Conservation status.

At present, there is limited information available to allow a conclusive determination of the conservation status of *Selaginella mucugensis*. Nevertheless, according to IUCN (2012) categories and criteria, we tentatively considered this species to be vulnerable (VU) on account that it is so far known from a single locality in the Espinhaço Mountain Range, which is threatened by human activities (Rapini et al. 2008).

#### Discussion.

*Selaginella mucugensis* is a member of subg. *Stachygynandrum* and may be confused with *Selaginella blepharodella* because they have similar leaf margins and indument on the upper surfaces in the distal region of median leaves and sporophylls (Fig. 7B). In fact, these two species may prove to be sympatric in the Serra do Sincorá, where both were collected. According to Harley and Simmons (1986), this area is an important center of diversity of the Brazilian montane flora. *Selaginella mucugensis* is distinguished from *Selaginella blepharodella* by the characters discussed under the diagnosis.

**Figure 10.**
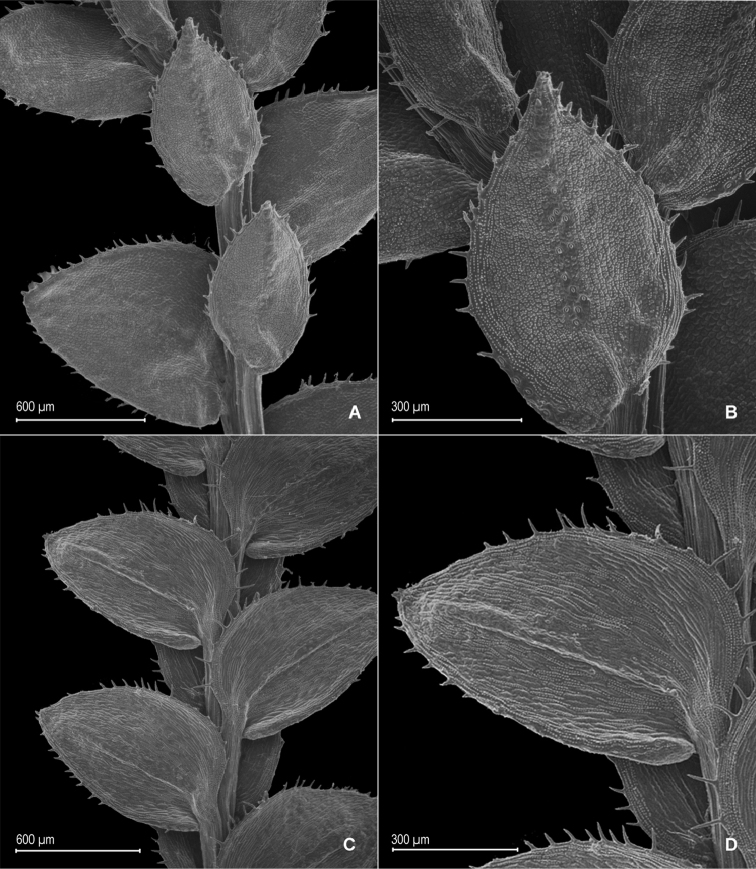
*Selaginella mucugensis* Valdespino. **A** Section of upper surface of stem **B** Upper surface of median leaf **C** Section of lower surface of stem **D** Lower surface of lateral leaf **A–D** taken from holotype, *Giulietti et al. [CFCR 1430]* (NY).

**Figure 11.**
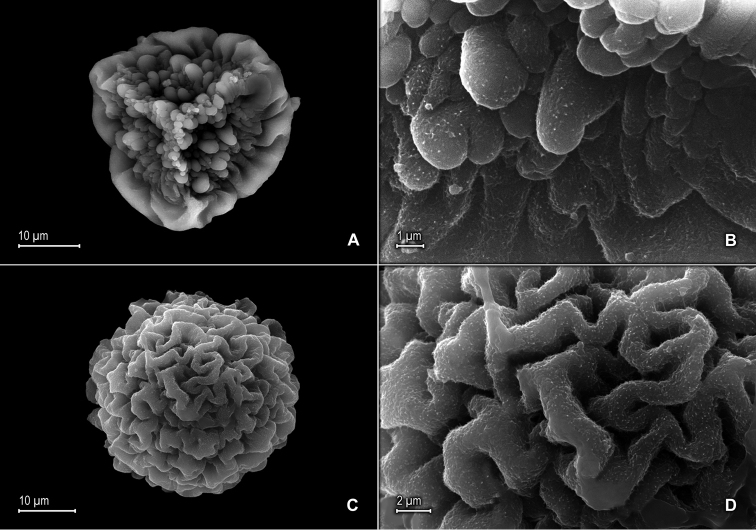
*Selaginella mucugensis* Valdespino. **A** Microspore proximal face **B** Close-up of microspore proximal face surface **C** Microspore distal face **D** Close-up of microspore distal face surface **A–D** taken from holotype, *Giulietti et al. [CFCR 1430]* (NY).

### Selaginella saltuicola

Taxon classificationPlantaeSelaginellalesSelaginellaceae

Valdespino sp. nov.

urn:lsid:ipni.org:names:77147603-1

Figures 12 , 13

#### Diagnosis.

*Selaginella saltuicola* is morphologically close to *Selaginella prasina* Baker but differs from it by having median leaves on main stems ovate or ovate-elliptic (vs. oblong to oblong-elliptic), with acute (vs. obtuse) apices, distally entire (vs. toothed), inner margins entire (vs. dentate distally), narrowly hyaline (vs. green) with (vs. without) a band of 1–3 elongate and papillate cells, leaf bases rounded to oblique (vs. decurrent), strobili borne throughout the stems and weakly defined (vs. terminal and compact), with (vs. without) continuous, vegetative growth from the apices, sporophylls similar to (vs. well-differentiated from) vegetative leaves, and light-orange (vs. deep orange) megaspores.

#### Type.

**BRAZIL**. Mato Grosso: Chapada dos Guimarães, Gorge of Véu de Noiva [ca. 15°24'21"S, 55°50'12"W], [ca. 720 m] 17 Oct 1973, *G.T. Prance et al. 19126* (holotype: NY!; isotypes: INPA!, PMA-fragment!).

#### Description.

*Plants* epipetric or epiphytic. *Stems* creeping, stramineous to green, 1.5–3 cm long, 0.05–0.2 mm diam., exarticulate, not flagelliform or stoloniferous, 1-branched. *Rhizophores* axillary, borne throughout stems, filiform, 0.05–0.1 mm diam. *Leaves* heteromorphic throughout, thin-membranaceous, both surfaces glabrous, upper surfaces green, lower surfaces silvery green. *Lateral leaves* distant or imbricate apically, patent to slightly ascending, ovate, ovate-elliptic or ovate-oblong, 0.9–1.5 × 0.5–0.8 mm; bases rounded, acroscopic bases slightly to strongly overlapping the stems, basiscopic bases free from the stems; acroscopic margins on upper surfaces greenish or narrowly hyaline, if the latter, in a band 1 or 2 cells wide, the cells elongate and papillate parallel to margins, papillae in 1 row over each cell lumen, on lower surfaces hyaline in a band 2–4 cells wide, the cells as on upper surfaces, entire or minutely serrulate along distal ¼, basiscopic margins on upper surfaces greenish on lower surfaces, narrowly hyaline marginally in a band 2–4 cells wide, the cells as along acroscopic hyaline margins, entire or inconspicuously denticulate throughout; apices rounded to broadly acute, entire or tipped by 1–3 teeth; upper surfaces comprising rounded to quadrangular, sinuate-walled cells, some of these on or near basiscopic and apical regions of the laminae covered by 2–4 papillae, without idioblasts and with stomata along margins, lower surfaces comprising elongate, sinuate-walled cells, some of these papillate and idioblast-like, papillae in 1 row over each cell lumen, with stomata irregularly distributed along midribs, as well as on acroscopic half of the laminae and on both margins (visible in both surfaces of the laminae). *Median leaves* distant or imbricate apically, ascending to spreading, ovate or ovate-elliptic, 0.6–0.9 × 0.4–0.5 mm; bases rounded or oblique, ventricose (i.e., swollen); margins narrowly hyaline in a band 1–3 cells wide, the cells elongate and papillate parallel to margins, papillae in 1 row over each cell lumen, entire; apices acute, entire (not distinctly tipped by teeth or cilia); both surfaces without idioblasts, upper surfaces comprising rounded to quadrangular, sinuate-walled cells, many of these covered by 2–4 papillae, with stomata throughout the laminae and some near submarginal region of the outer bases, lower surfaces comprising elongate, sinuate-walled cells, without stomata. *Axillary leaves* similar to lateral leaves. *Strobili* borne throughout the stems, weakly defined, lax, flattened, 1.0–2.0 mm. *Sporophylls* similar to or slightly differentiated from vegetative leaves, monomorphic to subdimorphic, without a laminar flap, ovate, 0.7–1.4 × 0.5–0.8 mm, each without a keel; bases rounded; margins narrowly hyaline, entire; apices acute, entire (not distinctly tipped by teeth or cilia); *dorsal sporophylls* with upper surfaces green and cells as in median leaves, except for the half that overlaps the ventral sporophylls, there hyaline to greenish hyaline with elongate, papillate, and slightly sinuate-walled cells, lower surfaces silvery green and comprising elongate, sinuate-walled cells; *ventral sporophylls* with both surfaces hyaline to greenish hyaline, comprising elongate, sinuate-walled cells. *Megasporangia* few in 1 ventral row; *megaspores* light-orange, mostly absent, proximal and distal faces not observed, not measured. *Microsporangia* in 2 dorsal rows and in 1 ventral row or few and in axils of median leaves; *microspores* deep orange, areolate-fossulate with granulate microstructure on proximal and distal faces, 25–31 µm.

#### Habitat and distribution.

*Selaginella saltuicola* is unique among other species here described by its apparent adaptation to very wet areas near waterfalls and perhaps even partially submerged in water along creek banks in Cerrado vegetation. At present, this species is known only from the high plateau of the Chapada dos Guimarães, Mato Grosso, Brazil, where it may be a local endemic, growing on wet rocks or wet logs at 600–720 m.

#### Etymology.

The epithet of the new species is derived from the Latin *saltus*, meaning jump, drop or fall and *cola*, meaning dweller, inhabitant, and alludes to it habitat near “cachoeiras” (waterfalls).

#### Conservation status.

*Selaginella saltuicola* seems to be restricted to the Chapada dos Guimarães area, where the Cerrado vegetation is dominant and severely threatened by human activities (Oliveira-Filho and Martins 1991, Ratter et al. 1997, Strüssmann and Mott 2009). *Selaginella saltuicola* may therefore be tentatively considered vulnerable (VU), according to IUCN (2012) categories and criteria, at least until additional distributional and conservation status studies can be carried out.

#### Additional specimens examined (paratypes).

**BRAZIL**. **Mato Grosso**: Waterfall at first Igarapé after descending Chapada on road to Cuiabá, 600 m, 23 Oct 1973, *Prance et al. 19336* (INPA, NY), *19337* (INPA, K, NY); Chapada dos Guimarães, Gorge of Véu de Noiva, 17 Oct 1973, *Prance et al. 19123* (INPA, NY), *19127* (NY), *19128* (INPA, NY), *19136* (INPA, NY), *19138* (NY).

#### Discussion.

**Figure 12.**
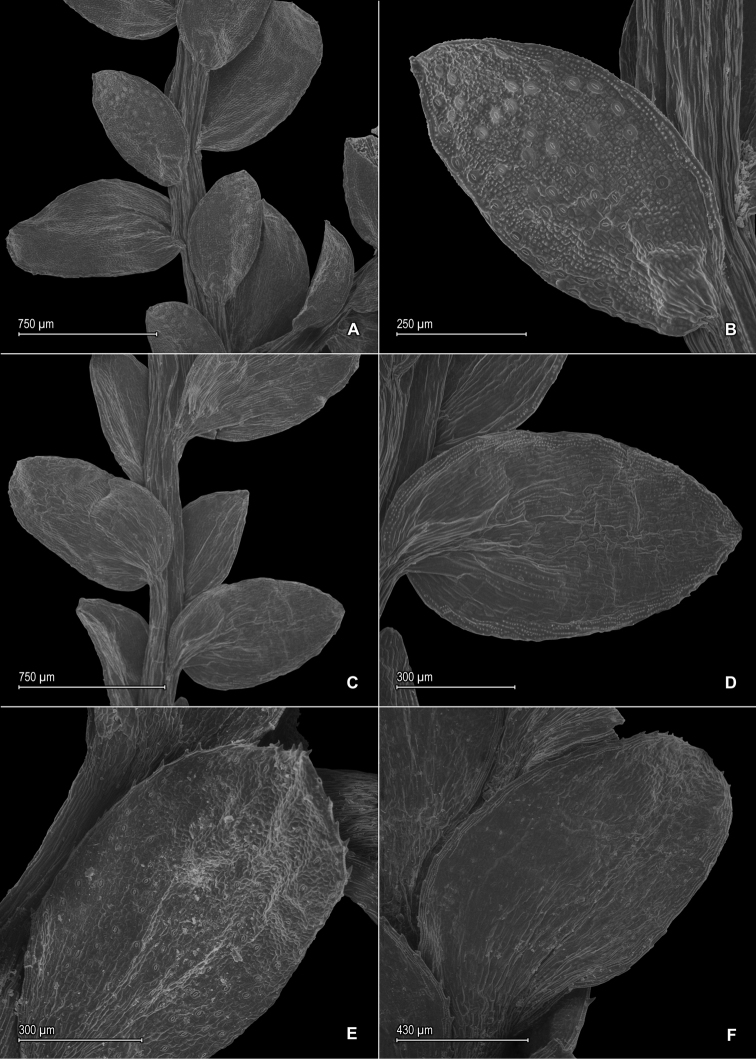
*Selaginella saltuicola* Valdespino. **A** Section of upper surface of stem **B** Upper surface of median leaf **C** Section of lower surface of stem **D** Lower surface of lateral leaf. **A–D** taken from paratype, *Prance et al. 19337* (NY). *Selaginella prasina* Baker **E** Upper surface of median leaf **F** Lower surface of lateral leaf **E**, **F** taken from *Smith et al. 115583* (GH).

*Selaginella saltuicola* belongs to subg. *Stachygynandrum* and is morphologically similar to *Selaginella prasina* from Cuba, *Selaginella salazariae* Valdespino from Panama, and *Selaginella undata* Shelton & Caluff, from Cuba, because they share similar habit and overall vegetative leaf morphology, stomata throughout upper surfaces of median leaves, and midribs of lateral leaves restricted to ca. ¼ below apices. However, *Selaginella undata* (isotype: *Shelton & Caluff 4514*, B!) falls within the morphological range of *Selaginella prasina* and may be best considered conspecific with the latter. *Selaginella saltuicola* differs from *Selaginella prasina* by the characters of median leaf shape, apex type, inner margin color and projections, leaf base shape, strobilus morphology, and megaspore color, as discussed in the diagnosis, as well as by having ovate, ovate-elliptic, or ovate-oblong (vs. obovate) axillary leaves and many cells on the upper surfaces of median leaves covered by 2–4 (Fig. 12B) [vs. without (Fig. 12E)] papillae. It differs from *Selaginella salazariae* in its median leaves ovate or ovate-elliptic (vs. obovate, obovate-elliptic, or broadly elliptic) with acute (vs. abruptly cuspidate to short-aristate) apices.

We note that Neotropical *Selaginella* species studied (i.e., *Selaginella prasina*, *Selaginella salazariae*, and *Selaginella saltuicola*) that grow either partially underwater or constantly wetted by waterfalls, rivers, or creeks have numerous stomata distributed over the upper surfaces of median leaves (Fig. 12A, B, E) and broadly acute to obtuse, rounded (Fig. 12A, C, D) or truncate lateral leaves (Fig. 12F). At present, it is not clear if the shared characters among those species might be the result of adaptation to a similar habitat (i.e., wet rocks or logs on waterfalls or stream banks) by convergent evolution or synapomorphies that may phylogenetically relate them.

In some plants of *Selaginella saltuicola*, as well as in some of *Selaginella alstonii*, we found strobili with continuous, vegetative growth from their apices. This condition was reported to occur in the genus by Hieronymus (1901), Williams (1931), Jermy (1990), and Valdespino (1993a, 1993b, 1995). In *Selaginella*, normally, fertile shoots (strobili) originate from the tips of vegetative shoots (i.e., stems and branches) in a “vegetative (V)/determinate fertile (F) growth pattern” or “V/F pattern,” although in plants of some species, e.g., *Selaginella decomposita* and *Selaginella saltuicola*, microsporangial development was observed in axils of median leaves, similarly to what is seen in *Selaginella denticulata* (L.) Spring where mega- and microsporangia are found in axils of lateral leaves below the weekly differentiated strobilus (see images in Quiles 2015). In the phenomenon described for *Selaginella saltuicola* and *Selaginella alstonii*, however, the fertile growth becomes indeterminate and the apices of strobili revert to a vegetative condition in what could be termed a “V/indeterminate F/V growth pattern” or “V/F/V pattern” that is also found in other species such as *Selaginella finitima* Mickel & Beitel, *Selaginella porphyrospora* A. Braun, and *Selaginella tenella* (P. Beauv.) Spring in mainland in the Neotropics (Valdespino 1995), *Selaginella orbiculifolia* Shelton & Caluff from Cuba (Caluff and Shelton 2003), and *Selaginella wangpeishanii* Li Bing Zhang, H. He & Q. W. Sun from China, which Zhang et al. (2014) termed TST (where T is for trophophyll = vegetative leaf, and S is for sporophyll) arrangement of microphylls. In a third condition, the second vegetative growth of the V/F/V pattern of the shoot becomes fertile and indeterminate in a “V/F/ V/indeterminate F growth pattern” or “V/F/V/F” pattern, found for example in *Selaginella correae* Valdespino from Panama (Valdespino 1993b), *Selaginella oregana* D.C. Eaton from temperate zones in western North America (Valdespino 1993a), and *Selaginella tuberculata* Spruce ex Baker (e.g., *Steyermark 75483*, NY!) from South America. This V/F/V/F pattern consists of a shoot with alternating vegetative leaves, sporophylls, and vegetative leaves along the stems and is reminiscent of the pattern found in some species of *Huperzia* (Lycopodiaceae). Valdespino (1995) suggested these alternating patterns of vegetative stems and fertile shoot formation could be an adaptive strategy of *Selaginella*, or it could be a response to damage to the growing apices. In any case, hormones may probably mediate this phenomenon, which seems to be more common and found across geographically and phylogenetically different *Selaginella* taxa than previously acknowledged. The ecological advantages of such variation, phylogenetic significance, and possible genetic and/or hormonal origin remain to be determined.

**Figure 13.**
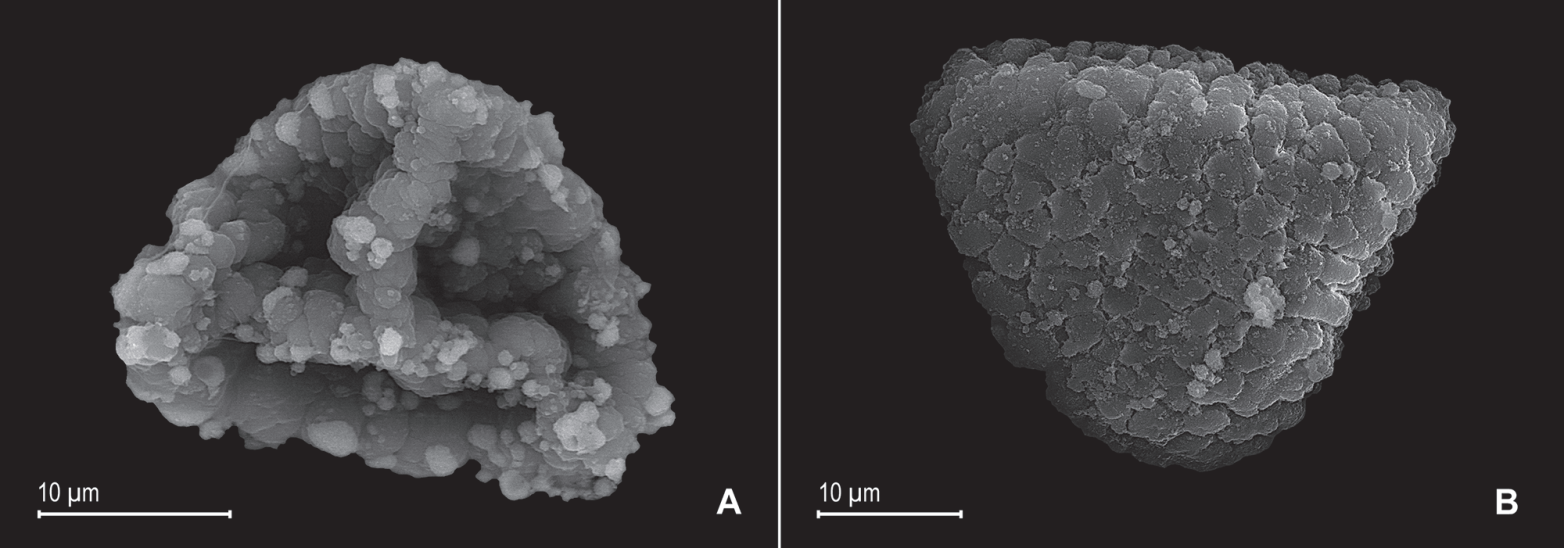
*Selaginella saltuicola* Valdespino. **A** Microspore proximal face **B** Microspore distal face **A, B** taken from holotype, *Prance et al. 19126* (NY).

### Selaginella sematophylla

Taxon classificationPlantaeSelaginellalesSelaginellaceae

Valdespino, G. Heringer & Salino sp. nov.

urn:lsid:ipni.org:names:77147604-1

Figures 14 , 15

#### Diagnosis.

*Selaginella sematophylla* differs chiefly from *Selaginella vestiens* in having (vs. lacking) idioblasts on upper surfaces of median leaves and dorsal sporophylls and on lower surfaces of lateral leaves, and by its acute (vs. acuminate to aristate, 0.4–0.7 mm) median leaves, and creeping (vs. ascending to suberect) habit.

#### Type.

**BRAZIL**. Minas Gerais: São Sebastião do Paraíso, Baú, [ca. 20°53'52"S, 46°57'33"W], 26 Apr 1945, *A.C. Brade & A. Barbosa 17953* (holotype: MO!; isotypes: BM!, CESJ!, NY!, PMA-fragment!, RB-image!).

#### Description.

*Plants* terrestrial. *Stems* creeping, stramineous, 2–3.5 cm long, 0.1–0.3 mm diam., exarticulate, not flagelliform or stoloniferous, 1-branched. *Rhizophores* axillary, borne throughout stems, filiform, 0.05–0.1 mm diam. *Leaves* heteromorphic throughout, thin-membranaceous to chartaceous, both surfaces glabrous, upper surfaces green, lower surfaces silvery green. *Lateral leaves* distant or imbricate distally, patent, ovate, 1.0–1.4 × 0.6–0.9 mm; bases rounded, acroscopic bases strongly overlapping the stems, basiscopic bases free from the stems; acroscopic margins hyaline to green-hyaline in a band 2–6 cells wide, the cells elongate and papillate parallel to margins, papillae in 1 row over each cell lumen, short- to long-ciliate along proximal ¼–½ and serrulate distally; basiscopic margins hyaline in a band 2 or 3 cells wide, the cells as on acroscopic margins, short-ciliate along proximal ⅕, otherwise serrate to serrulate along distal ⅘; apices acute, tipped by 1–3 teeth; upper surfaces comprising quadrangular to rounded, sinuate-walled cells, some of these on or near basiscopic and apical regions of the laminae, which are covered by 1–5 papillae, without idioblasts or stomata, lower surfaces comprising elongate, sinuate-walled cells, some of these papillate and idioblast-like, papillae in 1 row over each cell lumen, with stomata along midribs and few irregularly distributed over laminae. *Median leaves* imbricate, ascending, lanceolate, 0.8–1.4 × 0.3–0.7 mm; bases oblique to rounded, margins hyaline in a band 3–7 cells wide, the cells elongate and papillate parallel to margins, papillae in 1 row over each cell lumen, serrate to denticulate throughout; apices acute, tipped by 1 or 2 teeth; upper surfaces comprising rounded to quadrangular, sinuate-walled cells, most of these covered by 1–7 papillae, and some idioblast-like, papillate, elongate cells with papillae in 1 row over each cell lumen along both sides of the midribs, with stomata in 1 or 2 rows along midribs and a few irregularly distributed on proximal region of inner half of the laminae, lower surfaces comprising elongate, sinuate-walled cells, without stomata. *Axillary leaves* similar to lateral leaves. *Strobili* terminal on branch tips, lax, slightly quadrangular, 2.0–8.0 mm. *Sporophylls* monomorphic to slightly dimorphic, without a laminar flap, lanceolate, 1–1.4 × 0.5–0.8 mm, each without a keel; bases rounded; margins hyaline, serrulate; apices gradually acute, tipped by 1–3 teeth; *dorsal sporophylls* with both surfaces having idioblasts, upper surfaces green with cells as in median leaves, except for the half that overlaps the ventral sporophylls, there hyaline to greenish hyaline and with elongate, papillate, and slightly sinuate-walled cells, lower surface silvery green comprising elongate, sinuate-walled cells; *ventral sporophylls* with both surfaces hyaline and comprising elongate, sinuate-walled cells. *Megasporangia* in 2 ventral rows; *megaspores* cream or light-yellow, rugulate-reticulate with granulate-perforate microstructure on proximal faces, reticulate or reticulate-granular with granulate-echinulate and perforate microstructure on distal faces, 275–290 µm. *Microsporangia* in 2 dorsal rows; *microspores* orange, psilate marginally to rugulate towards the center with granulate microstructure on proximal faces, rugulate-cristate or cristate with broad baculate-like projections and granulate microstructure on distal faces, 28–40 µm.

#### Habitat and distribution.

*Selaginella sematophylla* is known from Minas Gerais, Espírito Santo, and Rio de Janeiro states in Brazil. It grows in Campos Rupestres and Atlantic Rainforest vegetation on sandy soil in shaded, wet places at 1000–1230 m.

#### Etymology.

The epithet of the new species derives from the Greek, *sema -tos*, meaning sign, flag, mark and *phyllon*, meaning leaf; this refers to the presence of conspicuous, hyaline idioblasts on upper leaf surfaces.

#### Conservation status.

The distributional range of *Selaginella sematophylla* encompasses three southeastern states of Brazil, but the vegetation types it inhabits are in peril; thus, we believe advisable to consider it vulnerable (VU), according to IUCN (2012) categories and criteria.

#### Additional specimens examined (paratypes).

**BRAZIL. Minas Gerais**: Arredores de São Sebastião do Paraíso, Apr 1945, *Brade et al. [Beta 109]* (R); Baú, 26 Apr 1949, *Brade 3461* (CESJ); Serra Nova, Rio Pardo de Minas, Parque Estadual de Serra Nova, 15°39'37,5"S, 42°45'53,7"W, 1000–1230 m, 13 Mar 2007, *Salino et al. 11734* (BHCB). **Espírito Santo**: Santa Maria do Jetibá, Garrafão, Pedra do Garrafão, 20°10'24,5"S, 40°55'6,8"W, 1081 m, 28 Aug 2009, *Salino et al. 14543* (BHCB, PMA). **Rio de Janeiro**: Santo Antônio do Imbé, Mandigueira, Apr 1932, *Brade & Santos-Lima 11670* (R).

#### Discussion.

**Figure 14.**
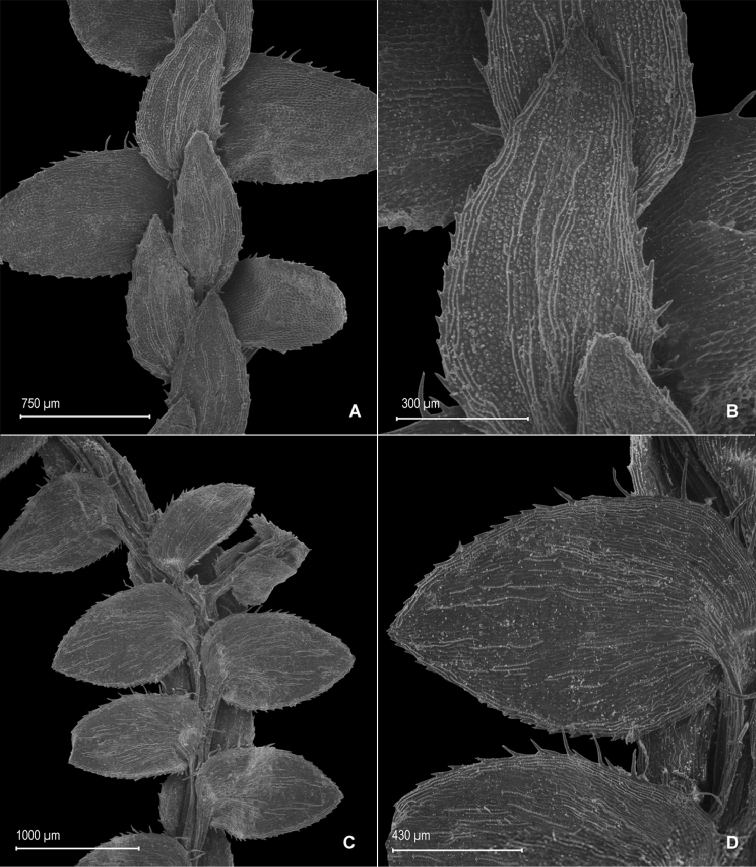
*Selaginella sematophylla* Valdespino, G. Heringer & Salino. **A** Section of upper surface of stem **B** Upper surface of median leaf **C** Section of lower surface of stem **D** Lower surface of lateral leaf **A–D** taken from paratype, *Brade et al. [Beta 109]* (R).

*Selaginella sematophylla* is a member of subg. *Stachygynandrum* and is characterized by having stems 1-branched, lateral and median leaves with hyaline margins, and idioblasts on upper surfaces of median leaves (Fig. 14A, B), lower surface of lateral leaves (Fig. 14C, D), and on both surfaces of sporophylls.

In the past, specimens of *Selaginella sematophylla* were identified as *Selaginella fragillima* (= *Selaginella vestiens*, which see for discussion). *Selaginella sematophylla* differs from *Selaginella vestiens* by cell types on leaf surfaces, median leaf apex shape, and habit, as discussed in the diagnosis.

**Figure 15.**
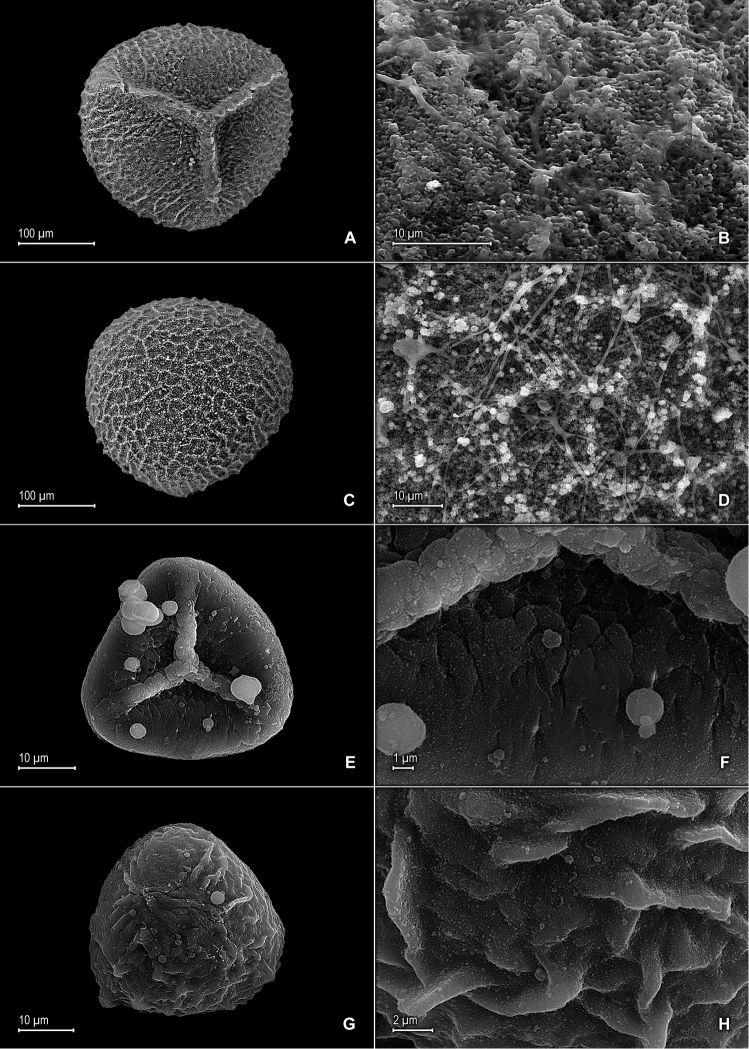
*Selaginella sematophylla* Valdespino, G. Heringer & Salino. **A** Megaspore proximal face **B** Close-up of megaspore proximal face surface **C** Megaspore distal face **D** Close-up of megaspore distal face surface **E** Microspore proximal face **F** Close-up of microspore proximal face surface **G** Microspore distal face **H** Close-up of microspore distal face surface **A–H** taken from holotype, *Brade & Barbosa 17953* (MO).

### Selaginella palmiformis

Taxon classificationPlantaeSelaginellalesSelaginellaceae

Alston ex Crabbe & Jermy, 1973

Selaginella palmiformis Alston ex Crabbe & Jermy, Amer. Fern J. 63: 141. 1973. - Type. Venezuela. Amazonas: Near Salto de Huá, in western foothills of Sierra Imeri, 800 m, *E. Holt & E. Blake 490* (holotype: US!; isotypes: BM! [photo: NY!, QCA!], NY!).
Selaginella manausensis Bautista, Bol. Mus. Paraense Emílio Goeldi, n.s., Bot. 45: 2. 1974. —Selaginella bahiensis Spring subsp. manausensis (Bautista) Jermy & Rankin, Bull. Brit. Mus. (Nat. Hist.) Bot. 9: 260. 1981. —Type: Brazil. Amazonas: Estrada Manaus-Itacoatiara, Km 64, picada I, 10 Oct 1968, *Rodrigues, Coêlho & Monteiro 8588* (holotype: INPA-image!; isotype: MG!). **Syn. nov.**

#### Additional specimens examined.

**COLOMBIA**. **Amazonas**: Corregimiento Araracuara, Caño Aduche, 30 Aug 1977, *Aguirre 907* (COL); Corr. Araracuara, Río Caquetá, 7 Aug 1977, *Idrobo 8939* (COL); La Pedrera, 2-3 km along E main road, 01°18'N, 69°32'W, 300 m, 10–11 Mar 1990, *Churchill 16094* (MO, NY); Río Caquetá, Cueva de los Guácharos, 250–500 m, 13 Aug 1979, *Echeverry 3364* (COL); Río Caquetá, cerca a Santa Isabel, 250 m, 26 Sep 1988, *Galeano & Miraña 1948* (COL). **Amazonas-Vaupés**: Río Apaporis, Raudal del Jirijirimo, 7 Aug 1951, *Schultes & Cabrera 13459* (BM, GH, MO, S, UC, US); Caño Oo-gö’-dja, Jenogojé, 26 Aug 1952, *Schultes & Cabrera 17058* (US). **Guainía**: Maimachi, Serranía del Naquén, Caño Culebra, 02°06'N, 68°11'W, 150 m, *Madriñan & Barbosa 822* (MO, NY); Río Guainía, Caño Guarinuma, 150 m, 10 Oct 1977, *Espina et al. 153* (COL). **Vaupés**: Río Guainía, near Sejal, June 1948, *Schultes & López 10162* (GH, MO); Río Kananarí, Cerro Isibukurí, 250-700 m, 28 Oct 1951, *Schultes & Cabrera 14465* (US); Río Piraparaná, 28 Aug 1952, *Schultes & Cabrera 17076* (UC, US); Río Piraparaná (tributary of Río Apaporis), Caño Teemeeña, 00°15'S-25'N, 70°30'W, 5 Sep 1952, *Schultes & Cabrera 17185* (GH p.p.), *Schultes & Cabrera 17190* (US), 10 Sep 1952, *Schultes & Cabrera 17369* (NY, US-2 sheets). **VENEZUELA**. **Amazonas**: Dpto. Atabapo, Alto Cunucunuma, 04°08'N, 65°35'W, 380 m, Feb 1992, *Chaviel 385* (NY), between Culebra and slope of Duida, 03°44'N, 65°44'W, 210 m, 16 Feb 1985, *Liesner 17568* (MO, NY, UC), camino entre Culebra y la falda del extremo N del Cerro Duida, SW of Comunidad de Culebra, 03°40'N, 65°45'W, 180–300 m, 28, 30 Jan and 1 Feb 1982, *Steyermark et al. 125726* (NY, UC); Río Cunucunuma, alrededores de Akanaña, 03°27'N, 65°44'W, 170 m, Apr 1990, *Fernández 7946* (MO-2 sheets, NY); Cerro Duida, base on N side opposite Culebra, 03°44'W, 65°44'N, 210–350 m, 10 Oct 1988, *Liesner 24640* (MO, NY, UC), slopes of Mount Duida, 750 ft [229 m], 15 Nov [1928?], *Tate 376* (NY), in saddle between Duida and Marahuaca near base of Duida, 03°34'N, 65°32'W, 1000 m, 25 Oct 1988, *Liesner 25363* (MO, NY, UC), slope of Huachamacari, 03°39'N, 65°42'W, 750 m, 6 Mar 1985, *Liesner 18382* (MO, UC), Cerro Huachamacarí, E slope, 03°49'N, 65°42'W, 600–700 m, 2 Nov 1988, *Liesner 25604* (MO, UC), Caño Negro, Río arriba desde la confluencia con Río Cunucunuma, 03°40'N, 65°45'W, 8 Feb 1982, *Steyermark 126269* (NY, UC); Río Cunucunuma, Río Orinoco, Playa Alta near river mouth, 100 m, 6 Nov 1950, *Maguire et al. 29452* (NY, US); Dpto. Río Negro, slopes of Cerro Aracamuni, 01°24'N, 65°38'W, 600 m, 21 Oct 1987, *Liesner & Delascio 22264A* (MO, NY, UC); Dpto. Río Negro, Neblina Base Camp on Río Bario (= Río Mawarinuma), SE of camp, 00°49'50"N, 66°09'40"W, 140 m, 27 Jan 1985, *Beitel & Buck 85065*, *Beitel & Buck 85066* (NY, UC), *Beitel & Buck 85067* (NY, UC). **PERU**. **Amazonas**: Dist. Bagua, Imaza, Aguaruna de Putuim, W of Putuim Village, 04°55'S, 78°19'W, 680 m, 12 Jun 1996, *Rodríguez et al. 968* (MO, NY), along road Imaza-Chiriaco, 05°03'24"S, 78°20'17"W, 400 m, 18 Mar 2001, *van der Werff et al. 16181* (MO). **Loreto**: Prov. Maynas, Dist. Iquitos, carretera del Caserio del Varillal, km 10, trail from Varillal, ca. 160 m, 4 Oct 1983, *Rimachi 7101* (NY). **BRAZIL**. **Acre**: Santa Lucia, km 40 on Transamazonica Highway E of Cruzeiro do Sul, 07°08'S, 72°33'W, 14 Oct 1987, *Pruski et al. 3466* (NY). **Amazonas**: Manaus, Rio Turumã, 23 Aug 1949, *Fróes 25063* (RB); Manaos [Manaus], Sep 1929, *Huebner 67* (B-2 sheets); Rio Cuieiras, 50 km upstream, 3 Apr 1974, *Campbell et al. P21811* (GH, K, MO, NY-2 sheets, R, S); Rio Urubú, between Serra da Lua and Iracema, 8 Aug 1979, *Calderón et al. 2978* (NY-2 sheets), between Cahoeira Iracema and Manaus-Caracarai Road, 6 Jun 1968, *Prance et al. 5017* (NY). **WITHOUT COUNTRY** [**BRAZIL**?]. *Bartlett s. n.* (W).

#### Discussion.

*Selaginella palmiformis* is a member of subg. *Stachygynandrum* and is characterized by its usually 1-pinnate branches that look like miniature palm leaves. According to Alston et al. (1981) this species was restricted to the Sierras of the Amazonian part of Venezuela and Colombia; however, Smith et al. (2005) recorded it in the Department of Loreto, Peru at 100–200 m and here we registered it in Amazonas Department of that country, where it was collected at 680 m. Both Departments are located in the Peruvian Amazon region. We also confirm the distribution range of *Selaginella palmiformis* to include the states of Acre and Amazonas in Brazil. It can be surmised that this species is widespread in the Amazon River basin in South America and that it grows in lowland tropical rainforests and in premontane wet forests from 100 to 1000 m.

Alston et al. (1981) considered *Selaginella manausensis* a subspecies of *Selaginella bahiensis* Spring (= Selaginella bahiensis subsp. manausensis). As part of his ongoing monographic work on the “*Selaginella flabellata* (L.) Spring group” the senior author studied the types of *Selaginella bahiensis* [BRAZIL. Bahia: In vicinia urbis Soteropoleos, *Blanchet 2528* (holotype: G!; isotypes: photo BM!, G!, P-2 sheets!], *Selaginella manausensis*, and *Selaginella palmiformis*. Based on this we conclude that *Selaginella manausensis* is not closely related to *Selaginella bahiensis* but rather it is conspecific with *Selaginella palmiformis* and, accordingly, it is synonymized here.

### Selaginella vestiens

Taxon classificationPlantaeSelaginellalesSelaginellaceae

Baker, 1883

Selaginella vestiens Baker, J. Bot. 21: 97. 1883. - *Selaginella cladostachya* Baker, J. Bot. 21: 97. 1883. - Type. Brazil. Goiás: Morro de Canto Gallo, *Burchell [7006]* (holotype: K!; isotype: B p.p.!).
Selaginella erythrospora A. Silveira, Bol. Commiss. Geogr. Geol. Est. Minas Geraes 5: 126. 1898. - Type: Brasil, Minas Geraes [Gerais], in rupibus, locis arenosis in Serra do Linheiro prope urbem S. João d’ El Rei, Apr 1897, *A. Silveira s.n.*, *No. 2383 in herb. Com. Geog. et Geolog. Civitatis Minas Geraes* (holotype: R! [*as Hebarium Silveira No. 156]*; isotypes: B!, BM! [*as Hebarium Silveira No. 156]*).
Selaginella fragillima A. Silveira, Bol. Commiss. Geogr. Geol. Est. Minas Geraes 5: 127. 1898. - Type: Brasil, Minas Geraes [Gerais], in umbrosis sub rupibus in Serra de S. José d’ El Rei prope Aguas Santas, Mar 1898, *A. Silveira s.n., No. 2622 in herb. Com. Geog. et Geolog. Civitatis Minas Geraes* (holotype: R! [as *Hebarium Silveira No. 149]*; isotypes: B!, P-image!). **Syn. nov.**

#### Selected specimens examined.

**BRAZIL**. **Goiás**: same as type coll. **Minas Gerais**, Belo Horizonte, 9 Jul 1932, *Brade 11881* (R); Biribyri, Mar 1892, *Schwacke 8028* (RB); Campos de S. Sebastião, Ouro Preto, Jun 1907, *Damazio 1882* (B-2 sheets, P-image, RB); Catas Altas, RPPN do Caraçá, 20°05'28"S, 43°29'00"W, 1500 m, 1 Jun 2008, *Hirai et al. 563* (NY, PMA, UC); Christias, near Corriga dois Puntes, Diamantiha [Diamantina], *Mexia 5832* (BM, CAS, GH, MICH, MO, S, U); Matta, Jun 1934, *Brade 13962* (RB); km 138, Estrada Pilar, Serra do Cipó, 15 Apr 1935, *Barreto 581 & Brade 144404* (RB); Santa do Riacho, Serra do Cipó, km 125 da Rodovia Belo Horizonte-Conceicão do Mato Dentro, 1320–1370 m, 29 Jun 1991, *Pirani et al. CFSC12385* (NY); Serra do Cipó, Jun 1908, *Damaizo s. n.* (RB); Serra de Ouro Preto, *Ule s. n.* (B); Serra do Rio Grande, 1260 m, Diamantiha [Diamantina], *Mexia 5799a* (CAS, GH, MO, NY, UC); Serra do Espinhaço, ca. 18 km E. of Diamantina, Diamantina, 1050 m, 20 Mar 1970, *Irwin et al. 27953* (NY), slopes of Serra da Piedade, ca. 35 km E of Belo Horizonte, near BR-31, 1800 m, 18 Jan 1971, *Irwin et al. 28699* (NY); Without specific locality, *Schwacke s. n.* (B).

#### Discussion.

*Selaginella vestiens* belongs to subg. *Stachygynandrum* and is characterized by its erect habit, stoloniferous stems, leaves seemingly monomorphic below first branch, and median leaves acuminate to aristate, ciliate, and broadly hyaline. *Selaginella fragillima* was a poorly known taxon that Alston et al. (1981) maintained as a distinct species. Our examination of type material of *Selaginella fragillima* causes us to conclude that it is conspecific with *Selaginella vestiens*, under which it is synonymized here. See comparison of *Selaginella vestiens* with *Selaginella sematophylla* under the latter.

Alston et al. (1981) cited *Ule 7298* (B!, BM!) from Bahia and *Glaziou 11723* (BM!, P-image!, US!) from Rio de Janeiro as *Selaginella vestiens*. *Ule 7298* is here assigned to *Selaginella blepharodella*, while *Glaziou 11723* morphologically does not fit *Selaginella vestiens*; therefore, we exclude Bahia and Rio de Janeiro from the range of the latter species. Specimens of *Selaginella vestiens* here cited and those cited by Heringer (2011) are either from Goiás (i.e., type collection) or from Minas Gerais in Brazil.

## Supplementary Material

XML Treatment for Selaginella alstonii

XML Treatment for Selaginella blepharodella

XML Treatment for Selaginella crinita

XML Treatment for Selaginella mucronata

XML Treatment for Selaginella mucugensis

XML Treatment for Selaginella saltuicola

XML Treatment for Selaginella sematophylla

XML Treatment for Selaginella palmiformis

XML Treatment for Selaginella vestiens
